# Identification and Computational Analysis of Novel *TYR* and *SLC45A2* Gene Mutations in Pakistani Families With Identical Non-syndromic Oculocutaneous Albinism

**DOI:** 10.3389/fgene.2020.00749

**Published:** 2020-07-21

**Authors:** Nousheen Bibi, Asmat Ullah, Lubna Darwesh, Waqas Khan, Tanzeela Khan, Kalim Ullah, Bushra Khan, Wasim Ahmad, Umm-e-Kalsoom

**Affiliations:** ^1^Department of Bioinformatics, Shaheed Benazir Bhutto Women University, Peshawar, Pakistan; ^2^Department of Biochemistry, Quaid-i-Azam University, Islamabad, Pakistan; ^3^Department of Molecular Biology, Shaheed Zulfiqar Ali Bhutto Medical University, Islamabad, Pakistan; ^4^Department of Biochemistry, Hazara University, Mansehra, Pakistan; ^5^Department of Zoology, Kohat University of Science and Technology, Kohat, Pakistan; ^6^Department of Biochemistry, Abdul Wali Khan University Mardan, Mardan, Pakistan

**Keywords:** dynamic simulation, molecular modeling, non-syndromic oculocutaneous albinism, tyrosinase (TYR), *SLC45A2*

## Abstract

Non-syndromic oculocutaneous albinism (nsOCA) is an inherited disorder of melanin biosynthesis with autosomal recessive mode of inheritance, presenting either hypopigmented or depigmented skin, hair, and eyes. It is genetically heterogeneous with seven loci (OCA1–OCA7) reported to date. In the present study, we have reported three consanguineous families (A, B, C) presenting identical nsOCA phenotypes. Sanger sequencing revealed a novel [NM_000372.5: c.826 T > C, p.(Cys276Arg)] and a recurrent variant [NM_000372.5: c.832C > T, p.(Arg278^∗^)] in tyrosinase (*TYR*) in families A and B, respectively. Microsatellite marker-based homozygosity mapping linked family C to OCA4. Sequence analysis identified a novel insertion variant (NM_016180.5: c.1331_1332insA) in the *SLC45A2*. Further, *in silico* mutagenesis and dynamic simulation approaches revealed that a novel Cys276Arg variant abolished the cysteine bridge and might contribute toward decreased stability of the TYR protein. Our study expands the mutation spectrum of the *TYR* and *SLC45A2* genes and emphasizes that molecular investigations are essential for accurate disease diagnosis.

## Introduction

Non-syndromic oculocutaneous albinism (nsOCA) is an inherited disorder of melanin biosynthesis with an autosomal recessive mode of inheritance. Skin, hair, and eyes in nsOCA patients are either hypopigmented or completely devoid of melanin (Shin Hae et al., 2012). The accompanying optic defects include poor visual acuity, photodysphoria, strabismus, nystagmus, foveal hypoplasia, iris transillumination, and abnormal projection of visual fibers (King et al., 1995).

The nsOCA is genetically heterogeneous with seven loci (OCA1–OCA7) reported to date. Among the seven forms, OCA1 (MIM 203100) is the most common and severe with a prevalence rate of 1 in 40,000 individuals worldwide and is caused by genetic defects in the tyrosinase (*TYR/*OCA1) located on chromosome 11q14–q21 (Supplementary Table S1). It codes for tyrosinase enzyme present inside the melanocytes of the skin, hair, and eyes (Cooksey et al., 1997; Gronskov et al., 2007). Tyrosinase is a copper-containing enzyme that catalyzes the first two steps of melanin biosynthesis. OCA1 is further categorized into two subtypes: OCA1A, where patients represent unpigmented hair and skin throughout life due to complete absence of tyrosinase activity, and OCA1B, where some of the enzyme activities are present, causing some melanin accumulation over time (Gronskov et al., 2007; Simeonov et al., 2013).

OCA2 (MIM 203200) caused by *OCA2* (previously called P; 15q11.2–q12) accounts for approximately 30% of the OCA cases worldwide. The encoded protein is a transmembrane protein that mediates a chloride-selective anion conductance required for melanin synthesis (Passmore et al., 1999; Bellono et al., 2014; Kamaraj and Purohit, 2014). OCA type3 (MIM 203290) is the most common type in black Africans affecting almost 1 in 8,500 individuals (Rooryck et al., 2008). It is caused by defects in *TYRP1* (9p23) that encodes tyrosinase-related protein 1 (TYRP1), which is involved in maintaining the melanosomal structure and affects the melanocyte proliferation and cell death (Kamaraj and Purohit, 2014).

OCA4 (MIM 606574) is rarely reported from Asian and European populations with a prevalence rate of 1 in 100,000, but it accounts for 24% of OCA cases in Japan (Inagaki et al., 2004). It is caused by defects in the *SLC45A2* (5p13.3) that encodes a solute carrier family 45 member 2 (SLC45A2) protein, which is present in melanosomal membranes and ensures the elevated pH in the melanosome by functioning as a proton/sugar symporter (Bin et al., 2015). The genetic identity of OCA5 locus mapped to chromosome 4q24 is yet to be discovered (Kausar et al., 2013a).

OCA6 (MIM 113750) and OCA7 (MIM 615179) are caused by defects in the solute carrier family 24 member 5 (*SLC24A5*) and *C10orf11*, respectively (Gronskov et al., 2013; Wei et al., 2013; Montoliu et al., 2014).

Several studies from Pakistan have reported molecular analysis of the nsOCA (Kausar et al., 2013a, b; Shah et al., 2015; Gul et al., 2017, 2019; Shahzad et al., 2017). Among the pathogenic variants detected so far, the *TYR* and *OCA2* are the most common causes of autosomal recessive OCA in the Pakistani population (Shahzad et al., 2017). OCA4 is rarely reported from the Pakistani population, with only four mutations reported to date (Kausar et al., 2013b; Shah et al., 2015). In the present study, we have reported three families (A–C) of Pakistani origin segregating nsOCA in an autosomal recessive manner. Affected individuals in all the three families had similar severe phenotypes, but molecular investigation revealed a novel (NM_000372.5: c.826 T > C; p.Cys276Arg) and recurrent variant (NM_000372.5: c.832C > T; p.Arg278^∗^) in *TYR* in families A and B, respectively, and a novel insertion variant (NM_016180.5: c.1331_1332insA; p.Asn444LysfsX5) in the *SLC45A2* in family C.

## Materials and Methods

### Family History

**FIGURE 1:**
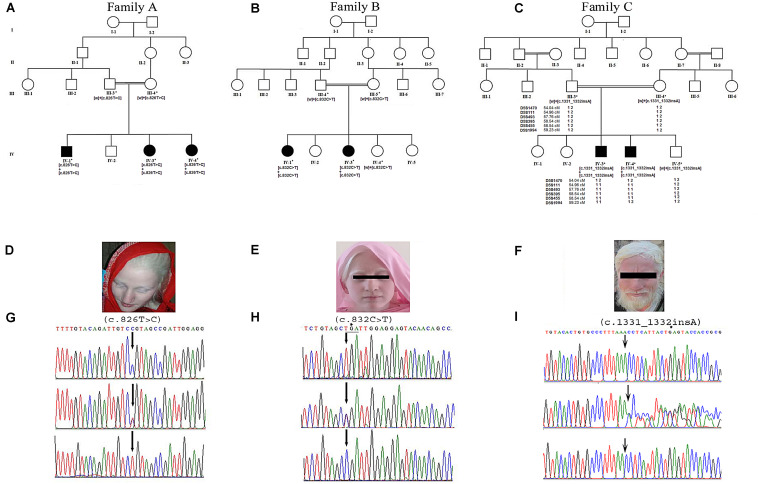
Panels **(A–C)** show pedigree drawings of the three families (A–C) segregating the disorder in an autosomal recessive manner. Individuals marked with asterisks (*) indicate the samples available for the study. In family C, the haplotype of microsatellite markers is shown below the symbols. Panels **(D–F)** represent photographs of affected members of the three families showing white milky skin and completely unpigmented hair, eyebrows, and eyelashes. **(G,H)** Upper panels represent the nucleotide sequence of a novel (c.826T > C) and a recurrent (c.832C > T) mutation identified in exon 2 of the *TYR* in families A and B, respectively. The middle and lower panels represent the nucleotide sequence in the heterozygous carriers and unaffected healthy individuals, respectively. **(I)** Upper panel represents nucleotide sequence (c.1331_1332insA) identified in the *SLC45A2* in family C. Middle panel shows the sequence in heterozygous carrier and the lower panel in healthy individual. Above the nucleotides in panels **(H,I)** represents the stop codon generated due to mutations.

Three consanguineous families, with autosomal recessive nsOCA, were recruited from Khyber Pakhtunkhwa, Pakistan. Evidence for the autosomal recessive mode of inheritance of the disorder was strongly supported by the pedigree drawings (Figures 1A–C). Affected members were carefully examined, and no additional phenotype other than OCA-related was observed. Blood samples were obtained from all available members including parents and affected and healthy siblings in all three families in EDTA vacutainer sets. The study was approved by the Departmental Review Committee and Ethical Review Committee of Hazara University, Mansehra, Pakistan. Written informed consent for collection of blood samples and publication of clinical and research data in peer-reviewed journals was obtained from all those who participated in the study.

### DNA Extraction and Molecular Investigation

Genomic DNA was extracted using DNA purification kit (Thermo Fisher Scientific, Lithuania) following the manufacturer’s guidelines. To assess the quality of extracted DNA, it was analyzed on 1% agarose gel followed by staining with ethidium bromide.

Based on the clinical findings recorded in affected members, the *TYR* (ENSG00000077498) was sequenced in all three families. Primers, to polymerase chain reaction (PCR)-amplify exons and splice junction sites of the gene, were designed using the Primer3 software. Primer sequences, melting temperature (Tm), and amplicon sizes are available on request. Initially, one affected member from each family was selected for sequencing *TYR*. PCR reaction was carried out in 50 μl volume containing 50 ng genomic DNA, 20 μl ready reaction mastermix, and 10 pmoles forward and reverse primers. The thermocycling conditions used included initial denaturation at 95°C for 5 min followed by 35 cycles at 94°C for 1 min, 57–59°C for 1 min, 72°C for 45 s, and final extension at 72°C for 10 min.

The amplified PCR products were analyzed on 2% agarose gel. Later, these were purified by commercially available kits (MBI Fermentas, Life Sciences, United Kingdom) prior to subjecting to sequencing. Analysis of the sequenced data was performed *via* BIOEDIT 7.0.9^1^.

In family C, after failing to detect any disease-causing variant in the *TYR*, homozygosity mapping was performed by typing microsatellite markers flanking genes causing other types of nsOCA. Physical and genetic distances of the markers were obtained from Rutger’s map built 36.2 (Matise et al., 2007). For PCR, markers were amplified using the standard conditions (Umm-e-Kalsoom et al., 2012) and resolved by electrophoresis on 8% non-denaturing polyacrylamide gel (PAGE). The *SLC45A2* (ENSG00000164175) was then sequenced by using the same conditions as described above.

Segregation of the identified mutations with the disorder within the families was carried out by sequencing the rest of the available affected and unaffected members. Polymorphism of the mutations identified in *TYR* and *SLC45A2* was excluded by searching their presence in various databases and screening 100 healthy, ethnically matched control individuals.

### *In silico* Analysis and Structural Predictions

Structural and functional influence of the identified variants was carried out through Polyphen^2^, SIFT^3^ and I-MUTANT 3.0 server^4^, PROVEAN-Protein Variation Effect Analyzer^5^, PhD-SNP-Predictor of human Deleterious Single Nucleotide Polymorphisms^6^ and SNPs&GO^7^, and Mutation Taster^8^.

Secondary structure details were retrieved through PDBsum^9^, and full-length three-dimensional (3D) structures of human TYR and SLC45A2 proteins were constructed through homology modeling approach^10^ using Swiss model^11^ and ITasser server (Yang and Zhang, 2015) using 5M8Q (44.4% identity) and 4M64 (87% identity) as template for TRY and SLC45A2, respectively. Stereochemistry and validity of the constructed 3D protein structures were assessed by Ramachandran plots (Laskowski et al., 1993), ProQ (Ray et al., 2012), and Verify3D (Eisenberg et al., 1997), and the coarse packing quality was evaluated with WHAT IF (Vriend, 1990). To enhance the model quality for further analysis, energy minimization and structure refinements were made using GROMMACS available in Chimera 1.5.6 (Pettersen et al., 2004) and VEGA ZZ^12^.

### *In silico* Site-Directed Mutagenesis and Root-Mean-Square Deviation Calculation

The 3D structure of the mutant (TYR^C276R^) was constructed using wild-type protein (TYR^WT^) as a template. The mutant model was evaluated for its stereo chemical quality and environmental profile using PROCHECK (Laskowski et al., 1996) and ERRAT (Structure analysis and verification servers), respectively. To minimize the potential energy and to calculate root-mean-square deviation (RMSD) of native and mutant structures, NOMAD-Ref^13^ and Chimera 1.5.6 tools were used (Pettersen et al., 2004).

### Hydropathy and Topology Analysis

To evaluate and differentiate the transmembrane (TM) helices of normal and mutated proteins, Membrane Protein Explorer (MPEx) was used to analyze the hydrophobicity of the respective amino acids (Spyropoulos et al., 2004; Snider et al., 2009). The MPEx tool predicts the TM region by using experimental values of hydrophobicity scales based on biological and physical analyses. Effect of particular mutation on TM helices is usually evaluated through this tool. Additionally, TransMembrane protein Re-Presentation in 2 Dimensions (TMRPres2D) tool was used for 2D visualization of TM segments. Next, to investigate the arrangement of protein secondary structure to form pores and channels for passage of ions, CAVER analyte 2.0 was used (Chovancova et al., 2012).

### Molecular Dynamic Simulations

Molecular dynamic (MD) simulation studies of TYR^WT^ and TYR^C276R^ were made to assess the folding, stability, conformational changes, and dynamic behaviors of TYR. Amber03 force field embedded in GROMACS 4.5 package running on high-performance OpenSuse linux system was used to perform simulations (Duan et al., 2003). Throughout the simulation experiments, both TYR^WT^ and TYR^C276R^ systems were solvated by TIP4P water model in a periodic box (Zlenko, 2012). The system was neutralized by addition of Na^+^ and Cl^–^ counter ions. Energy minimization (steepest descent algorithm for 500 steps) was executed by tolerance of 1,000 kJ/mol Å^2^ to eliminate initial steric clashes. After completing the minimization steps, systems were subjected to simulations for 30 ns timescale under constant temperature (300 K) and pressure (1 atm). To this end, electrostatic interactions were calculated using Particle Mesh Ewald (PME) algorithm. To investigate the stability behavior of TYR^WT^ and TYR^C276R^ systems, VMD (Humphrey et al., 1996), PyMol^14^, and GROMACS tools were used.

## Results

### Clinical Description

Family A: three affected individuals (IV-1, IV-3, IV-4) in this family were aged 7–18 years at the time of the study. White milky skin, white hair, white eyebrows, and white eyelashes, indicating complete absence of pigment, were observed in all three affected members (Figure 1D). In two of the affected individuals (IV-1, IV-3), ophthalmic features including photophobia, nystagmus, and strabismus were recorded. However, iris color was gray/blue in all three individuals.

Family B: at the time of the study, affected members IV-1 and IV-3 were 13 and 3 years old, respectively. Both presented white skin, white hair, white eyebrows, and white eyelashes (Figure 1E). Elder affected sister (IV-1) had photophobia, nystagmus, and strabismus. Her eyesight was extremely weak, and she sits near a teaching board in the classroom. The strabismus was the only observable feature in the younger affected sister (IV-3). However, both of them had gray/blue iris color.

Family C: at time of the study, affected members IV-3 and IV-4 were 24 and 26 years of age, respectively. Both had white milky skin and depigmented hair, eyebrows, and eyelashes (Figure 1F). The ophthalmic features including nystagmus, strabismus, photophobia, and blue iris color were observed in both affected members. Eyesight was weak, and they were unable to see without glasses.

Unaffected individuals in all three families exhibited normal pigmentation of skin, hair, and eyes.

### Molecular Analysis

Sequence analysis of *TYR* revealed a novel mutation [c.826T > C, p.(Cys276Arg)] and a previously reported mutation [c.832C > T, p.(Arg278^∗^)] in affected members in families A and B, respectively. Both the mutations were present in homozygous state in all the affected members of two families (Figures 1G,H). In family C, after establishing the linkage at OCA4 on chromosome 5p13.3 (Figure 1C), sequence analysis of the candidate gene *SLC45A2* revealed a novel homozygous insertion mutation (c.1331_1332insA) in affected members (Figure 1I). The mutation led to a frameshift and premature termination codon 9 bp downstream in the same exon (p.Asn444LysfsX5). Various bioinformatics tools including SIFT, POLYPHEN, I-MUTANT, PROVEAN, PhD-SNP, SNPs&GO, and Mutation Taster predicted that the mutations, p.(Cys276Arg) and p.(Asn444LysfsX5), carry a damaging effect on structure and function of the TYR and SLC45A2 protein (Table 1).

**TABLE 1:** *In silico* Analysis of Mutations in *TYR* and *SLC45A2*.

		**Polyphen**	**Sift**	**I mutant**	**PROVEAN**	**PHD-SNP**	**SNPs&GO**	**Mutation taster**
TRY^Cys276Arg^	Score	1	0.00	RI = 6	−9.11	4	RI = 8	180
	Predicted effect	Deleterious	Damaging	Disease	Deleterious	Disease	Disease	Altered protein

SLC45A2^Asn444LysfsX5^	Score	0.7	0.05	RI = 4	−6.01	2	RI = 6	94
	Predicted effect	Deleterious	Damaging	Disease	Deleterious	Disease	Disease	Splice site change

*RI, reliability index.*

Unaffected healthy individuals in all three families were heterozygous for the identified mutations. None of the mutations was detected in 100 ethnically matched, unrelated, and healthy control individuals. Also, the identified mutations had negligible frequencies, if any, when searched in gnomAd in the general population^15^ (Table 2).

**TABLE 2:** Clinical and molecular investigation of affected individuals with mutations in *TYR* and *SLC45A2*.

**Gene**	***TYR***					***SLC45A2***	
**Family**	**A**			**B**		**C**	
Inheritance	AR			AR		AR	
Nucleotide change	c.826T > C			c.832C > T		c.1331_1332insA	
Amino acid change	p.(Cys276Arg)			p.(Arg278*)		p.(Asn444LysfsX5)	
AllelicFrequency * Wt/Mut	826T/C (0)			832C (0.999)/T (0.001)		1331A/AA (0)	
Affected members	IV-1	IV-3	IV-4	IV-1	IV-3	IV-3	IV-4
Gender	M	F	F	F	F	M	M
Age in years	15	13	7	13	3	24	26
Hair color	White	White	White	White	White	White	White
Eyebrows/eyelashes	White	White	White	White	White	White	White
Skin color	Milky white	Milky white	Milky white	Milky white	Milky white	Milky white	Milky white
Iris color	Gray/blue	Gray/blue	Gray/blue	Gray/blue	Gray/blue	Gray/blue	Gray/blue
Photophobia	+	+	−	+	−	+	+
Nystagmus	+	+	−	+	−	+	+
Strabismus	+	+	−	+	+	+	+
Eyesight	Weak	Weak	NA	Weak	NA	Weak	Weak

AR, autosomal recessive; Wt, wild type; Mut, mutant; M, male; NA, not available; + indicates the presence of a phenotypic character; − indicates the absence of a phenotypic character; * indicates frequencies of the variants, if any, found in gnomAd in the general population (https://gnomad.broadinstitute.org/).

### Secondary and Tertiary Structure of TYR and SLC45A2

**FIGURE 2:**
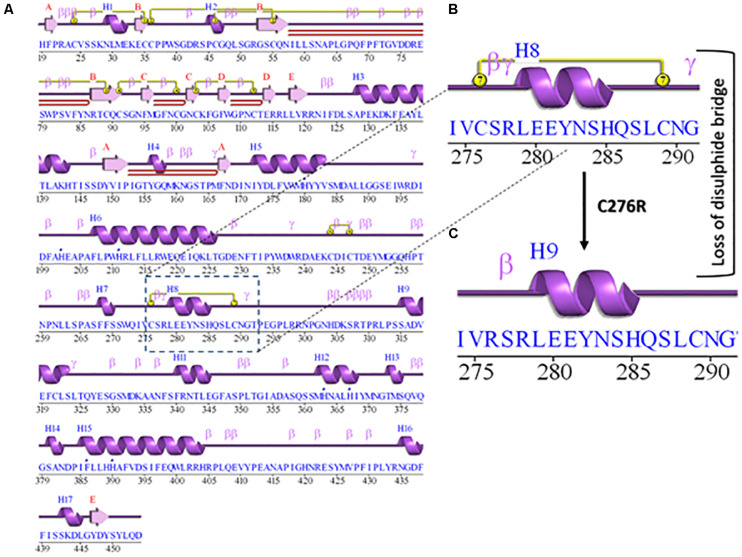
Comparative 2D structure and topology analysis of TYR^WT^ and TYR^C276R^. **(A)** Secondary structure of TYR^WT^, disulfide bridges are shown in yellow line, and β-hairpin structure is indicated by a red loop. **(B)** Close-up view of cysteine bridge of TYR^WT^ and **(C)** loss of cysteine bridge in TYR^C276R^.

**FIGURE 3:**
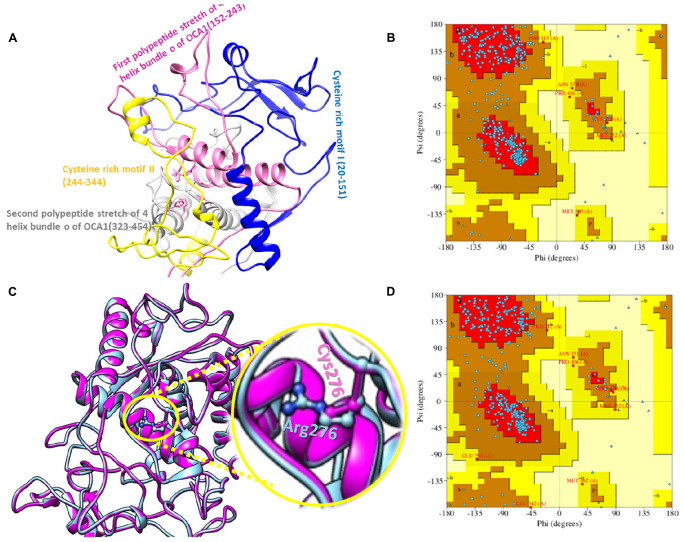
Structure validation and motif organization of human TYR protein. **(A)** Crystal structure of TYR is shown in ribbon model with its respective color depicting critical function. For each subunit, the different domains/motifs are identified on ribbon models and their respective function is indicated. **(B)** Ramachandran plot of TYR^WT^. **(C)** Superimposed model of TYR^WT^ (deep pink) and TYR^C276R^ (blue) while the wild type and mutant residues are shown in ball and stick model. **(D)** Ramachandran plot of TYR^C276R^.

The 2D structure analysis of TYR showed 18 alpha helices, 12 beta sheets, 7 disulfide bridges and four beta hairpins (Figures 2A,B). The novel p.(Cys276Arg) mutation identified in family A resulted in a loss of disulfide bridge (Figure 2C). Due to unavailability of the full-length TYR structure, homology modeling and protein threading techniques were used to model the TYR^WT^. The TYR^WT^ structure was used to model TYR^C276R^ that mapped Cys276Arg mutation in the cysteine-rich motif 2 (residues 244–322) of catalytic domain of TYR (Figure 3A). Normal and mutant TYR protein models were superimposed to find the model fluctuation. Both models were very well superimposed except the mutant residue (Figure 3B). The predicted models of TYR^WT^ and TYR^C276R^ were used to calculate the bond angles of amino acid coordinated using Ramachandran plot, which indicated approximately 97.37% residues in the allowed region. Furthermore, additional factors comprising of non-bonded interactions, peptide bond planarity, main chain H-bond energy, poor rotamers, overall G-factor, and Ca tetrahedral distortion for the modeled structures were mapped in the favorable range (Figures 3C,D).

**FIGURE 4:**
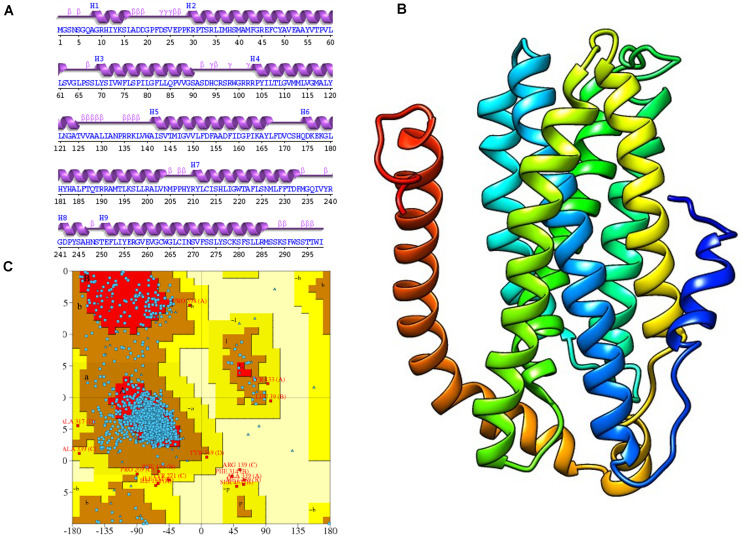
Protein modeling and its validation. **(A,B)** Secondary structure and tertiary protein model of SLC45A2. **(C)** Ramachandran plot of normal SLC45A2.

Secondary structure of SLC45A2 showed 11alpha helices (Figure 4A). Tertiary protein model of SCL45A2 was also constructed through homology modeling and threading approaches (Figure 4B). The resulting model was checked for stereochemistry and geometry optimization. Ramachandran plot indicated 98% residues in the favorable region with a z-score of −2.38 (Figure 4C). The RMSD between TYR^WT^ and TYR^C276R^ was 0.149, while the RMSD between SLC45A2^WT^ and SLC45A2^Asn444LysfsX5^ was 0.183, respectively.

### Assessment of Conservation Pattern of Cys276 in TYR

The functionally important amino acids are highly conserved. Therefore, to cross validate the evolutionary significance of a novel mutation p.(Cys276Arg) in the human TYR protein, multiple-species sequence alignment was performed using ClustalW with seven homologs. Results showed conservation of the cysteine at amino acid position 276 in the cysteine-rich motif 2 of TYR (Supplementary Figure S1A). Additionally, the change from polar uncharged amino acid to basic amino acid was clearly evident in 2D and 3D conformations (Supplementary Figures S1B,C).

### Hydropathy and Topology Plot Analysis

**FIGURE 5:**
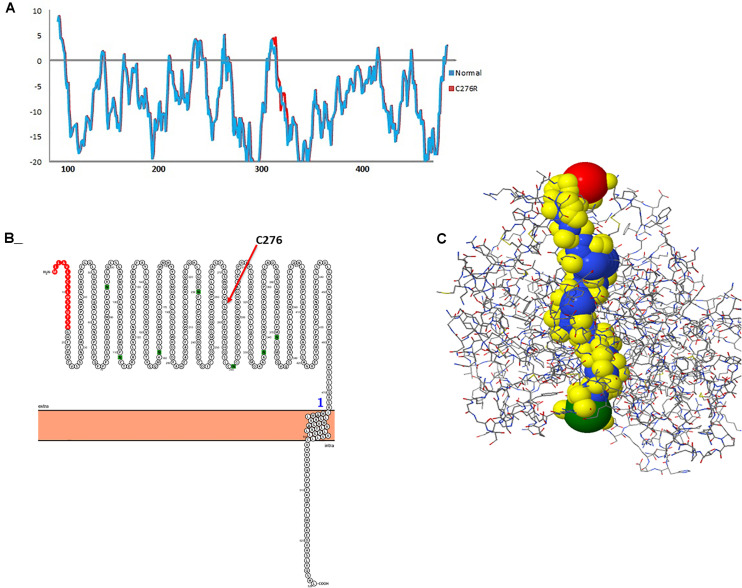
Hydropathy and topology analysis of TYR^WT^ and TYR^C276R^. **(A)** Hydropathy plot of normal and mutated TYR amino acids performed by the Membrane Protein Explorer (MPEx) tool. **(B)** Topology plot of TYR. Signal peptide is shown in red balls, while C276 residue is highlighted by a red arrow. **(C)** Tunnel in TYR protein shown in hydrophobic model and the rest of the protein in wire model. In hydrophobic model residues are colored according to their physiochemical properties.

To gauge the functional influence of p. (Cys276Arg) mutation in TYR, initially, we mapped and characterized its structural placement. Hydropathy results showed that the N-terminal part of the protein lies toward the extracellular part of the membrane, and the C-terminal part of TYR has TM helices. Hydropathy plot (Figure 5A) analysis showed that Cys276Arg substitution is significant for the structure of protein. Membrane protein functioning depends on its topology confirmation. The topology showed that the signal peptide at the N-terminus was extracellular and C-terminal region was intracellular. Overall, the topology was the same in wild-type and mutant TYR with minor shifting (Figure 5B). Protein tunnels help in connecting the functional buried cavities with bulk solvent and protein channels and facilitate the movement of ions and second messengers across the membrane.Interestingly, the Cys276Arg was observed in tunnel formation (Figure 5C), and Cys to Arg substitution within the tunnel might decrease the stability and activity of the TYR protein. Overall, pore length was 72.259 with a score of 0.844. Bottle neck of pore was 1.587 and straightness 0.849.

Next, the topology analysis of SLC45A2 showed 12 TM domains of native protein while the last two TM domains were deleted due to frameshift and premature stop codon in the mutant SLC45A2 protein (Supplementary Figure S2).

### Molecular Dynamics Simulation Analysis

**FIGURE 6:**
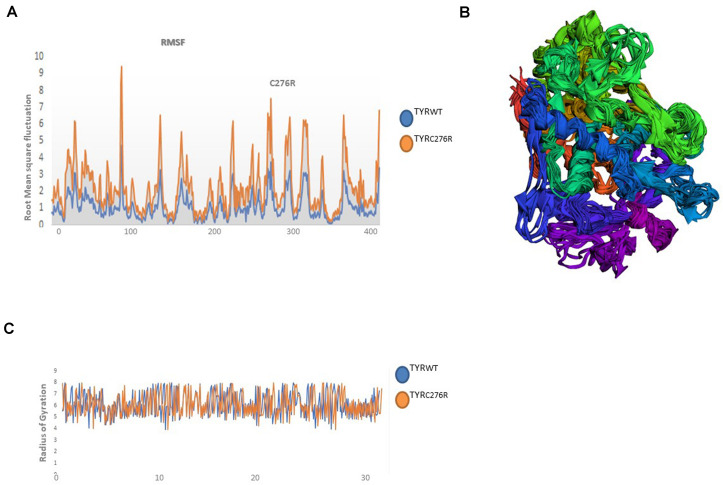
Plots to investigate the stability and fluctuation of molecular dynamic (MD) trajectories for a wild-type and mutant TYR. **(A)** Root mean square fluctuation (RMSF) plots computed through each system trajectory **(B)** Three-dimensional (3D) fluctuation of all trajectories on 30 ns timescale. **(C)** Radius of gyration (Rg) plot, TYR^WT^ (blue) and TYR^C276R^ (orange).

The TYR^WT^ and TYR^C276R^ were further analyzed by MD simulation assay in order to study the time-dependent behavior and to investigate the overall stability of the system. Atom trajectories were used to plot the root mean square fluctuation (RMSF) to measure the secondary structural elements’ stability and conformational deviations. In TYR^WT^, the high fluctuation was observed between 200 and 300 residues, and the system remained stable throughout the protein length. To our surprise, a region encompassing Cys276Arg indicated more fluctuations up to 5A° (Figure 6A). Fluctuation in this particular region was due to disulfide bridging of cysteine residues with arginine with different topologies. These data indicated less tight packaging of TYR^C276R^ as compared to TYR^WT^ (Figure 6B). The stability of secondary structure elements and conformational changes of TYR^WT^ and TYR^C276R^ systems were calculated by plotting the radius of gyration (Rg) obtained throughout the simulated trajectories. The Rg plot is the measure of stability and firmness of the system; the calculated Rg plot (Figure 6C) showed stability except a slight change at 4–5 ns depicting conformational changes upon unfolding.

## Discussion

In the present study, we have clinically and genetically investigated three consanguineous families (A, B, C) demonstrating the autosomal recessive form of nsOCA. Affected individuals in all three families presented similar cutaneous and ophthalmic features (Table 2). Sequence analysis of the candidate genes revealed three disease-causing variants. This included a novel missense p.(Cys276Arg) and a previously reported nonsense mutation p.(Arg278^∗^) in the *TYR* in families A and B, respectively, and a novel frameshift mutation p.(Asn444LysfsX5) in the *SLC45A2* in family C.

A novel missense mutation p.(Cys276Arg) in the *TYR*, identified in family A, involved substitution of a cysteine with arginine at amino acid position 276. Cysteine is one of the polar uncharged amino acids, which stabilizes the protein 3D structure through formation of a disulfide bridge. Human tyrosinase contains 17 cysteine residues distributed among two cysteine-rich motifs. Ten of these residues are located in the cysteine-rich motif I (20–151 residues) and the other five in the cysteine-rich motif II (244–322) (Simeonov et al., 2013). The substituted cysteine in p.(Cys276Arg) is present in motif II. Through *in silico* deep structural analysis, we have elucidated the structural and functional behavior of TYR upon cysteine substitution in motif II. In addition, homology modeling and threading-based approaches predicted the model structure of native and mutant TYR. The results showed substitution of Cys276 with Arg resulted in a loss of a disulfide bridge that existed between Cys35 and Cys276, which altered the conformation and configuration of the protein, leading to reduced protein stability. Hydropathy and topology analysis revealed that the mutation p.(Cys276Arg) is significantly pushing the helical region of the protein toward the intramembrane side. MD simulation analysis revealed that this mutation caused an alteration in protein structural behavior. In addition, fluctuations were observed in the loop region encompassing the mutation, and this alteration might play a role in inducing albinism in family A. The recurrent c.832C > T mutation in family B introduces the premature stop codon p.(Arg278^∗^) leading to TYR inactivation that lacks the second copper-binding domain.

*SLC45A2* was reported for the first time in Turkish OCA patients (Suzuki and Tomita, 2008). It has seven exons that encode the SLC45A2 protein also called membrane-associated transport protein (MATP), with 12 putative transmembrane domains. SLC45A2 is specifically expressed in melanosomes and endosomes. It functions as a sugar transporter by utilizing the proton gradient and maintains the melanosomal pH that is essential for copper ion coordination into the TYR. Small interfering RNA (siRNA)-mediated knockdown of SLC45A2 resulted in acidification of melanosomes that in turn reduced the l-3,4-dihydroxyphenylalanine (L-DOPA) oxidase activity of TYR and melanin content (Bin et al., 2015).

Mutations in *SLC45A2* are infrequently reported from Asian, European, African, and North Americans. Of the 95 mutations reported so far in *SLC45A2* (Supplementary Table S2), only four, including three novel and one recurrent mutation, have been reported from Pakistan (Kausar et al., 2013b; Shah et al., 2015). In this study, we have reported the fourth novel mutation (c.1331_1332insA) in the *SLC45A2* from Pakistan. The c.1331_1332insA mutation results in premature termination, leading to loss of the last two TM domains in the SLC45A2 protein. As our patients show the complete loss of pigmentation that is unusually reported for OCA4 phenotypes, so we could speculate that the truncated protein generated will most probably be destroyed by nonsense mediated decay (NMD). This may have affected the melanosomal activities to such an extent that even minimal melanin has not been synthesized.

## Conclusion

Conclusively, molecular and *in silico* analyses of identified mutations in TYR and SLC45A2 protein provide the pathogenicity prediction as well as structural and functional changes that are likely to confirm the contribution to pathogenesis of albinism. The present computational analysis provides a theoretical basis for the molecular mechanism of albinism underlying *TYR* and *SLC45A2* gene mutations. Our study expanded the mutation spectrum in the *TYR* and *SLC45A2* and emphasizes that molecular investigations are essential for accurate disease diagnosis. Overall, this study will contribute to further studies of disease prevention and treatment of TYR- and SLC45A2-related albinism.

## Data Availability Statement

The datasets for this article are not publicly available due to concerns regarding participant/patient anonymity. Requests to access the datasets should be directed to the corresponding author.

## Ethics Statement

Written informed consent was obtained from the individuals legal guardian for the publication of any potentially identifiable images or data included in this article.

## Author Contributions

U-K and WA contributed to the conception, design and supervision of the study. AU, LD, and WK performed the experimental work including DNA extraction, PCR, and gene sequencing. NB and TK performed the computational analysis. AU wrote the first draft of the manuscript. NB and BK wrote the sections of the manuscript. KU studied the families and collected the samples. WA critically reviewed the manuscript. All authors read and approved the submitted version.

## Conflict of Interest

The authors declare that the research was conducted in the absence of any commercial or financial relationships that could be construed as a potential conflict of interest.

## Footnotes

1 https://www.mybiosoftware.com/alignment/1013

2 http://genetics.bwh.harvard.edu/pph2

3 https://sift.bii.a-star.edu.sg/

4 http://www.cmbi.ru.nl/hope

5 http://provean.jcvi.org/index.php

6 http://snps.biofold.org/phd-snp/phd-snp.html

7 http://snps.biofold.org/snps-and-go/snps-and-go.html

8 http://www.mutationtaster.org/

9 https://bio.tools/pdbsum_generate

10 http://www.rcsb.org

11 https://swissmodel.expasy.org

12 http://www.ddl.unimi.it

13 http://lorentz.dynstr.pasteur.fr/nomad-ref.php

14 http://www.pymol.org

15 https://gnomad.broadinstitute.org/
