# Supplementary material for: Identification and Computational Analysis of Novel TYR and SLC45A2 Gene Mutations in Pakistani Families With Identical Non-syndromic Oculocutaneous Albinism
Source: Front Genet. 2020 Jul 21;11:749. doi: 10.3389/fgene.2020.00749 (PMC7385404; doi:10.3389/fgene.2020.00749)
Supplement: Supplementary file 1 [file Data_Sheet_1.docx]

**Supplementary Information**

**
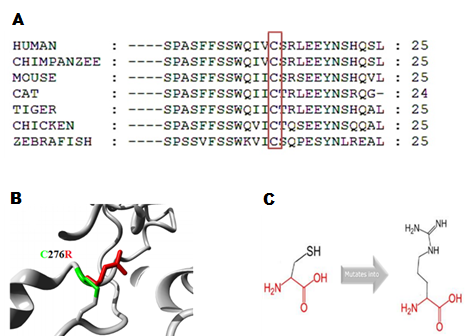
**

**Figure S1 Multiple sequence alignment (MSA) and** **Functional influence of C276R mutation in TYR**. **(A)** Showing the conservation of cysteine at amino acid position 276 across the closed homologues of TYR. **(B)** Effect of C276R substitution on protein structure. The protein is colored grey, the side chains of both the wild type (TYR^WT^) and the mutant (TYR^C276R^) residue are shown in green and red color respectively. **(C)** 2-D structure of wild type (TYR^WT^) and mutant (TYR^C276R^) tyrosinase protein.

**
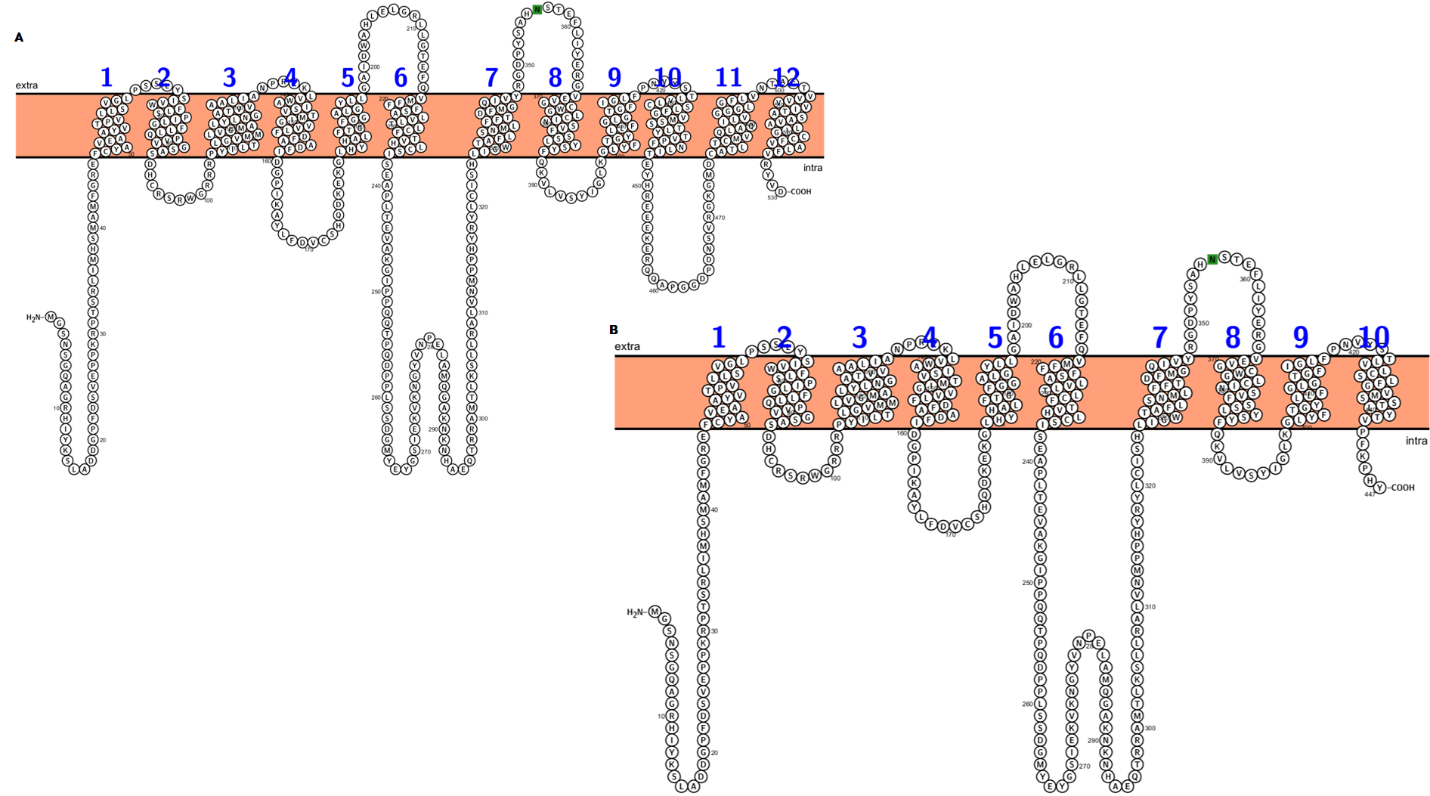
**

**Figure S2 Topology analysis of SLC45A2** **(A)** 12 transmembrane domains of wild type protein. **(B)** deletion of last two transmembrane domains due to frame shift and premature stop codon in mutant SLC45A2 protein.
