# Supplementary material for: Identification and Computational Analysis of Novel TYR and SLC45A2 Gene Mutations in Pakistani Families With Identical Non-syndromic Oculocutaneous Albinism
Source: Front Genet. 2020 Jul 21;11:749. doi: 10.3389/fgene.2020.00749 (PMC7385404; doi:10.3389/fgene.2020.00749)
Supplement: Supplementary file 2 [file Data_Sheet_2.docx]

**Table S1: Known *TYR* Mutations reported to date**

| **No** | **cDNA** | **Protein** | **Ethnicities** | **References** |
| --- | --- | --- | --- | --- |
| 1 | c.1A>G | p.Met1Val | Italian, British, Danish | [Breimer et al., 1994](http://www.ifpcs.org/albinism/oca1mut.html#bre94); [King et al., 2003](http://www.ifpcs.org/albinism/oca1mut.html#kin03); [Hutton and Spritz, 2008a](http://www.ifpcs.org/albinism/oca1mut.html#hut08); [Hutton and Spritz, 2008b](http://www.ifpcs.org/albinism/oca1mut.html#hut08b) |
| 2 | c.2T>C | p.Met1Thr | Caucasian | Oetting et al., 1993 |
| 3 | c.25delC | frame shift | Caucasian | Oetting et al., 1993, Spritz et al., 1997, Hutton and Spritz, 2008b |
| 4 | c.26T>C | p.Leu9Pro | African American | King et al., 2003 |
| 5 | c.32G>A | p.Trp11Ter | NA | Simeonov et al., 2013 |
| 6 | c.47delC | frame shift | Caucasian | Hutton and Spritz, 2008a |
| 7 | c.52delG | frame shift | Caucasian, Northern European | [Oetting et al., 1998](http://www.ifpcs.org/albinism/oca1mut.html#oet98); [King et al., 2003](http://www.ifpcs.org/albinism/oca1mut.html#kin03) |
| 8 | c.57T>A | p.His19Gln | Caucasian | [Oetting et al., 1998](http://www.ifpcs.org/albinism/oca1mut.html#oet98) |
| 9 | c.56A>G | p.His19Arg | Caucasian, Asian | Rooryck et al., 2008 |
| 10 | c.61C>T | p.Pro21Ser | Caucasian | [Tripathi, et al., 1992a](http://www.ifpcs.org/albinism/oca1mut.html#tri92a); [King et al., 2003](http://www.ifpcs.org/albinism/oca1mut.html#kin03); [Hutton and Spritz, 2008b](http://www.ifpcs.org/albinism/oca1mut.html#hut08b) |
| 11 | c.62C>T | p.Pro21Leu | Pakistani | Jaworek et al., 2012 |
| 12 | c.70T>C | p.Cys24Arg | Chinese | Wei et al., 2010 |
| 13 | c.71G>A | p.Cys24Tyr | Chinese | Wang et al., 2009 |
| 14 | c.73G>T | p.Val25Phe | German | Opitz et al., 2004 |
| 15 | c.74_75insT | frameshift | Caucasian | King et al., 2003, Opitz et al., 2004 |
| 16 | c.86A>C | p.Asn29Thr | German | Opitz et al., 2004 |
| 17 | c.98A>C | p.Lys33Thr | Turkish | Rooryck et al., 2008 |
| 18 | c.103T>C | p.Cys35Arg | Pakistani | Jaworek et al., 2012 |
| 19 | c.107G>A | p.Cys36Tyr | German | Passmore et al., 1999 |
| 20 | c.110C>T | p.Pro37Leu | Not reported | Summer et al., 2014 |
| 21 | c.115T>C | p.Trp39Arg | Chinese | Wei et al., 2010 |
| 22 | c.116G>A | p.Trp39Term | Italian | King et al., 2003 |
| 23 | c.117G>T | p.Trp39Cys | Chinese | Wei et al., 2010 |
| 24 | c.121G>A | p.Gly41Arg | Caucasian, Chinese | King et al., 2003 |
| 25 | c.124G>A | p.Asp42Asn | Eastern Indian (West Bengal) | Chaki et al., 2006 |
| 26 | c.124delG | frameshift | Caucasian | Hutton and Spritz, 2008b |
| 27 | c.125A>G | p.Asp42Gly | Caucasian northern European | King et al., 1991a; King et al., 2003 |
| 30 | c.130A>C | p.Ser44Arg | German | Opitz et al., 2004 |
| 31 | c.130A>G | p.Ser44Gly | German | Opitz et al., 2004 |
| 32 | c.131G > A | p.Ser44Asn | Japanese | Okamura et al., 2014 |
| 33 | c.132T>A | p.Ser44Arg | Pakistani | Shah et al., 2015 |
| 34 | c.133C>A | p.Pro45Thr | Caucasian | Hutton and Spritz, 2008a |
| 35 | c.137G>A | p.Cys46Tyr | Italian | Gargiulo et al., 2011 |
| 36 | c.139G>T | p.Gly47Cys | German | Opitz et al., 2004 |
| 37 | c.139G>A | p.Gly47Ser | Iranian | Khordadpoor-Deilamani et al., 2015 |
| 38 | c.140G>A | p.Gly47Asp | Caucasian, Hispanic, Moroccan Jew, canary islands, Puerto Rican, Cuban, Mexican, native American, German, Ashkenazi Tunisian Sephardic Jew | Oetting 1991a; Oetting et al., 1993; Gernochi-baruch et al., 1994; King et al., 2003; Opitz et al., 2004; Hutton and Spritz, 2008b |
| 39 | c.149C>G | p.Ser50Ter | Arab Christian | Gershoni-baruch et al., 1994 |
| 40 | c.149C>A | p.Ser50Ter | Lebanese | Zahed et al., 2005 |
| 41 | c.149C>T | p.Ser50Leu | Not reported | Simeonov et al., 2013 |
| 42 | c.151G>C | p.Gly51Arg | Not reported | Ray et al., 2007 |
| 43 | c.155G>T | p.Arg52Ileu | Caucasian, Korean | Oetting et al., 1998; Park et al., 2012 |
| 44 | c.155G>A | p.Arg52Lys | Not reported | Ray et al., 2007 |
| 45 | c.155G>C | p.Arg52Thr | Not reported | Ray et al., 2007; Urtatiz et al., 2014 |
| 46 | c.163 T>G | p.Cys55Gly | South America | Urtatiz et al., 2014 |
| 47 | c.164G>A | p.Cys55Tyr | Caucasian A | King et al., 1991a; Hutton and Spritz 2008a; Hutton and Spritz, 2008b |
| 48 | c.168G>T | p.Gln56His | in dbSNP(submit by CGAP-GAI) | Albinism Database |
| 49 | c.204A>T | p.Gln68His | German | Opitz et al., 2004 |
| 50 | c.216delA | p.T72Tfs* | Chinese | Lin et al., 2014 |
| 51 | c.223delG | frameshift | Caucasian | Oetting et al., 1998 |
| 52 | c.228C>G | p.Asp76Glu | Danish | Gronskov et al., 2009 |
| 53 | c.229C>T | p.Arg77Trp | Caucasian | Spritz et al., 1997; King et al., 2003, Hutton and Spritz, 2008b |
| 54 | c.229C>G | p.Arg77Gly | Chinese | Takeda et al., 1990; Wei et al., 2010 |
| 55 | c.230G>A | p.Arg77Gln | Japanese, Korean, Chinese, German | Takeda et al., 1990; Park et al., 1997; Tanita et al., 2002; King et al., 2003, Opitz et al., 2004; Wang et al., 2009; Shah et al., 2015 |
| 56 | c.232_233insGGG | p.Arg77_Gln78insG | Chinese | Tsai et al., 1999; Wang et al., 2009 |
| 57 | c.232G>T | p.Glu78Term | Caucasian | Spritz et al., 1997; Hutton and Spritz, 2008b |
| 58 | c.235T>C | p.Ser79Pro | Caucasian | King et al., 2003 |
| 59 | c.236C>T | p.Ser79Leu | German | Opitz et al., 2004 |
| 60 | c.238T>C | p.Trp80Arg | Caucasian | Spritz et al., 1997; King et al., 2003; Hutton and Spritz, 2008b |
| 61 | c.240G>A | p.Trp80Term | Caucasian | Oetting et al., 1998 |
| 62 | c.241C>T | p.Pro81Ser | Caucasian | King et al., 2003 |
| 63 | c.242C>T | p.Pro81Leu | Caucasian, German | Giebel et al., 1990; King et al., 2003; Opitz et al., 2004; Hutton and Spritz, 2008a; Hutton and Spritz, 2008b |
| 64 | c.248T > G | p.Val83Gly | Pakistani | Gul et al., 2019 |
| 65 | c.250T>G | p.Phe84Val | Caucasian | Hutton and Spritz, 2008b |
| 66 | c.255T>G | p.Tyr85Term | Lebanese | Zahed et al., 2005 |
| 67 | c.255T>A | p.Tyr85Term | Italian, Lebanese | Gargiulo et al., 2011 |
| 68 | c.265T>C | p.Cys89Arg | African American | Spritz et al., 1991 |
| 69 | c.265T>A | p.Cys89Ser | Iranian | Ghodsinejad Kalahroudi et al., 2014 |
| 70 | c.269A>G | p.Gln90Arg | in dbSNP (submit by TOMITA) | Albinism database |
| 71 | c.272G>C | p.Cys91Ser | eastern Indian (west Bengal) | Miyamura et al., 2005 |
| 72 | c.272G>A | p.Cys91Tyr | Hutterites | Chong et al., 2012 |
| 73 | c.286_287insA | frameshift | Caucasian | Oetting et al., 1991b; King et al., 2003; Hutton and Spritz, 2008b |
| 74 | c.283T > C | p.phe95Leu | Japanese | Okamura et al., 2014 |
| 75 | c.287T>A | p.Met96Asn | Caucasian | Oetting et al., 1991c |
| 76 | c.289G>A | p.Gly97Arg | Caucasian | Oetting et al., 1998 |
| 77 | c.290G>T | p.Gly97Val | Japanese | Goto et al., 2004 |
| 78 | c.299G>T | p.Cys100Phe | Chinese | Wei et al., 2010 |
| 79 | c.300T>G | p.Cys100Trp | Chinese | Wei et al., 2010 |
| 80 | c.307T>C | p.Cys103Arg | Chinese | Wang et al., 2018 |
| 81 | c.316G>C | p.Gly106Arg | Northern European | King et al., 2003 |
| 82 | c.325G>A | p.Gly109Arg | Caucasian | Camand et al., 2001 |
| 83 | c.338_339delCA | frameshift | Caucasian | Camand et al., 2001; Passmore et al., 1999; Hutton and Spritz, 2008b |
| 84 | c.344_345delGA | p.Arg115fsX52 | Pakistani, Caucasian, German | Oetting et al., 1993; King et al., 2003; Opitz et al., 2004; Hutton and Spritz, 2008b |
| 85 | c.346C>T | p.Arg116Term | Caucasian, Chinese, German | Oetting et al., 1998; King et al., 2003; Opitz et al., 2004; Wang et al., 2009; Gul et al., 2017 |
| 86 | c.368T>C | p.Ile123Thr | Caucasian | Hutton and Spritz, 2008b |
| 87 | c.373G>T | p.Asp125Tyr | in dbSNP (submit by TOMITA) | Albinism database |
| 88 | c.374G>A | p.Asp125Gly | Caucasian | Preising et al., 2011 |
| 89 | c.391A>G | p.Ly131Glu | Indian | Miyamura et al., 2005 |
| 90 | c.401T>G | p.Phe134Cys | in dbSNP (submit by SNP500) | Albinism Database |
| 91 | c.419T>G | p.Leu140Term | Not reported | Saxena and Verma, 2010 |
| 92 | c.425A>T | p.Lys142Met | Chinese | Wang et al., 2009 |
| 93 | c.426G>T | p.Lys142Asn | in dbSNP (submit by CGAP-GAI) | Albinism Database |
| 94 | c.445_446delTA | frameshift | Japanese | Ito et al., 2006 |
| 95 | c.446A>G | p.Tyr149Cys | Caucasian | Hutton and Spritz, 2008b |
| 96 | c.452T>G | p.Ieu151Ser | Chinese | Lin et al., 2011 |
| 97 | c.454C>T | p.Pro152Ser | Ashkenazi Jew | Gershoni-baruch et al., 1994 |
| 98 | c.455C>A | p.Pro152His | Not available | Liu et al., 2015 |
| 99 | c.459_460insT | frameshift | Caucasian (German) | Passmore et al., 1999 |
| 100 | c.463A>T | p.Thr155Ser | German | Opitz et al., 2004 |
| 101 | c.488C>G | p.Ser163Term | French | Rooryck et al., 2008 |
| 102 | c.505_507delGAC | p.Asp169del | English | Rooryck et al., 2008 |
| 103 | c.526T>A | p.F176Ile | Caucasian | Oetting and King, 1994 |
| 104 | c.529G>T | p.Val177Phe | German | Opitz et al., 2004 |
| 105 | c.530T>A | p.Val177Asp | Indian | Ray et al., 2007; Chaki et al., 2011 |
| 106 | c.533G>A | p.Trp178Term | Afghan | Giebel et al., 1991a |
| 107 | c.535A>T | p.Met179Leu | German | Opitz et al., 2004 |
| 108 | c.538C>A | p.His180Asn | German | Opitz et al., 2004 |
| 109 | c.539A.G | p.His180Arg | Iranian | Ghodsinejad Kalahroudi et al., 2014 |
| 200 | c.542A>G | p.Tyr181Cys | Caucasian | Hutton and Spritz, 2008b |
| 201 | c.551C>G | p. ser184Term | South America | Urtatiz et al., 2014 |
| 202 | c.549_550delGT | (p. V184fsX8) * | Chinese | Wang et al., 2015 |
| 203 | c.553A>G | p.Meth185Val | Chinese | Wei et al., 2010 |
| 204 | c.560_561ins25 bp | gross insertion | Chinese | Liu et al., 2014 |
| 205 | c.561_562insTTATTATGTGTCAAATTATCCCCCA | Frameshift; G190Cfs*12 | Chinese | Yang et al., 2019 |
| 206 | c.572delG | frameshift | Caucasian | Oetting et al., 1991b; King et al., 2003; Hutton and Spritz, 2008b |
| 207 | c.573delA | frameshift | Caucasian, Syrian | King et al., 2003 |
| 208 | c.575C>A | p.Ser192Tyr | Pakistani | Shah et al., 2015 |
| 209 | c.580delA | p.I209SfsX32 | South American | Urtatiz et al., 2014 |
| 210 | c.589_590delGA | frameshift | Caucasian | King et al., 2003 |
| 211 | c.593 T>C | p.Ile198Thr | Pakistani | Shah et al., 2014 |
| 212 | c.595G>A | p.Asp199Asn | German | Opitz et al., 2004 |
| 213 | c.601G>T | p.Ala201Ser | German | Opitz et al., 2004 |
| 214 | c.601delG | p.Ala201fsX24 | German | Opitz et al., 2004 |
| 215 | c.605A>G | p.His202Arg | Caucasian | Hutton and Spritz, 2008b |
| 216 | c.606T>G | p.His202Gln | Italian | Gargiulo et al., 2011 |
| 217 | c.613C>A | p.Pro205Thr | Caucasian European, Armenian | Camand et al., 2001; King et al., 2003; Hutton and Spritz, 2008b |
| 218 | c.616G>A | p.Ala206Thr | Caucasian | King et al., 1991a; Oetting and King, 1994 |
| 219 | c.626C>G | p.Pro209Arg | Caucasian | King et al., 2003 |
| 220 | c.626C>T | p.Pro209Leu | Caucasian | Hutton and Spritz, 2008b15 |
| 221 | c.632A>G | p.His211Arg | Chinese | Wei et al., 2010 |
| 222 | c.635G>C | p.Arg212Thr | Chinese | Wei et al., 2010 |
| 223 | c.635G>A | p.Arg212Lys | Caucasian | King et al., 2003 |
| 224 | c.640_642delTTC | p.Phe214del | Chinese | Liu et al., 2010 |
| 225 | c.646T>A | p.Leu216Met | Canary Islands | Oetting et al., 1993b, Oetting and king, 1994 |
| 226 | c.649C>G | p.Arg217Gly | Caucasian | Spritz et al., 1997; Hutton and Spritz, 2008b |
| 227 | c.649C>T | p.Arg217Trp | Caucasian | Tripathi et al., 1992; Oetting and king, 1994; King et al., 2003; Hutton and Spritz, 2008b |
| 228 | c.649delC | frameshift | Ashkenazi/Tunisian/Sephardic Jew, Caucasian | Gershoni-baruch et al., 1994; King et al., 2003; Hutton and Spritz, 2008b |
| 229 | c.650G>A | p.Arg217Gln | Caucasian European | King and Oetting, 1992; Oetting and king, 1994; King et al., 2003; Hutton and Spritz, 2008a; Hutton and Spritz, 2008b |
| 230 | c.649_650CG>TC | p.Arg217Ser | German | Opitz et al., 2004 |
| 231 | c.652T>C | p.Trp218Arg | Not reported | Saxena and Verma, 2010 |
| 232 | c.654G>A | p.Trp218Term | Arabian | Khan et al., 2016 |
| 233 | c.655G>A | p.Glu219Lys | southern Indian (Andhra Pradesh) | Chaki et al., 2006 |
| 234 | c.655G>T | p.Glu219Term | French | Rooryck et al., 2008 |
| 235 | c.661G>A | p.Glu221Lys | Caucasian | King et al., 2003 |
| 236 | c.665T>C | p.Ile222Thr | in dbSNP (submit by SNP500) | Albinism Database |
| 237 | c. 674T>C | p.Leu225Pro | Not reported | Summer et al., 2014 |
| 238 | c.678_680delAGG | p.Gly227del | Caucasian | Camand et al., 2001 |
| 239 | c.680_682delGAG | p.Gly227del | Netherlands | Gronskov et al., 2009 |
| 240 | c.703T>C | p.Tyr235His | Chinese | Wei et al., 2010 |
| 241 | c.706T>C | p.Trp236Arg | Chinese | Wei et al., 2010 |
| 242 | c.707G>A | p.Trp236Term | African | Oetting et al., 1993b |
| 243 | c.707G>C | p.Trp236Ser | Caucasian | Passmore et al., 1999 |
| 244 | c.707G>T | p.Trp236Leu | German | Opitz et al., 2004 |
| 245 | c.710delA | frameshift | Caucasian | Simeonov et al., 2013 |
| 246 | c.714G>A | p.Trp238Term | Chinese | Wei et al., 2011 |
| 247 | c.715C>T | p.Arg239Trp | Japanese, Chinese | Nakamura et al., 2002; King et al., 2003; Shah et al., 2015 |
| 248 | c.716G>A | p.Arg239Gln | in dbSNP (submit by SNP500) | Albinism Database |
| 249 | c.719A>T | p.Asp240Val | German | Opitz et al., 2004 |
| 250 | c.721G>A | p.Ala241Thr | Chinese | Lin et al., 2014 |
| 251 | c.728A>C | p.Lys243Thr | German | Opitz et al., 2004 |
| 252 | c.731_732delGT | Frameshift | Caucasian French | King et al., 2003; Hutton and Spritz, 2008b |
| 253 | c.732_733delTG | p.Cys244Term | Caucasian | Oetting et al., 1991b; Oetting et al., 1993 |
| 254 | c.739T>C | p.Cys247Arg | South American | Urtatiz et al., 2014 |
| 255 | c.746A>G | p.Asp249Gly | Chinese | Wei et al., 2010 |
| 256 | c.748G>T | p.Glu250Term | French | Aquaron et al., 2009 |
| 257 | c.757G>A | p.Gly253Arg | Israeli Arab | Spritz et al., 1997 |
| 258 | c.758G>A | p.Gly253Glu | Caucasian | King et al., 2003 |
| 259 | c.763C>T | p.Gln255Term | Danish | Gronskov et al., 2009 |
| 260 | c.766C>T | p.His256Tyr | Caucasian, German, Lebanese | Camand et al., 2001; Opitz et al., 2004; Zahed et al., 2005 |
| 261 | c.779C>T | p.Pro260Leu | in dbSNP (submit by CSHL-HAPMAP) | Albinism Database |
| 262 | c.781_784delAACT | frameshift | Cameroonian | Badens et al., 2006 |
| 263 | c.796G>A | p.Ala266Thr | in dbSNP (submit by APPLERA) | Albinism Database |
| 264 | c.796G>C | p.Ala266Pro | Israeli | Rosenmann et al., 2009 |
| 265 | c.814T>C | p.W272R | Lebanese | Zahed et al., 2005 |
| 266 | c.816G>C | p.W272C | Caucasian (German), Jordanian | Passmore et al., 1999 |
| 267 | c.816G>T | p.W272C | Not reported | Preising et al., 2011 |
| 268 | c.819G>T | p.Gln273His | Chinese | Sun et al., 2018 |
| 269 | c.820-1_820GA>TG | p.I274_S277>PfsX23 | French | Rooryck et al., 2008 |
| 270 | c.820-3C>G | splice mutation | Chinese, Taiwanese | Tsai et al., 1999; King et al., 2003; Hutton and Spritz, 2008b |
| 271 | c.820-2A>G | splice site mutation | Not reported | Simeonov et al., 2013 |
| 272 | c.820-2delA | splice mutation | German | Opitz et al., 2004 |
| 273 | c.823G>T | p.Val275Phe | Caucasian | Giebel et al., 1991c; King et al., 2003; Hutton and Spritz, 2008b |
| 274 | c.824-?_1007+?del | large deletion | French | Rooryck et al., 2008 |
| 275 | c.826 T>C | p.Cys276Arg | Pakistani | This study |
| 276 | c.827G>A | p.Cys276Tyr | Danish, Indian | Gronskov et al., 2009; Mondal et al., 2012 |
| 277 | c.832C>T | p.Arg278Term | indo-Pakistani, Moroccan Jew, Japanese, European, Mexican, Indian, Syrian, eastern Indian, Korean | Tripathi et al., 1993; Spritz, 1993; Gershoni-baruch et al.,1994; Tanita et al., 2002; King et al., 2003; Goto et al., 2004; Sundaresan et al., 2004; Chaki et al., 2006; Wang et al., 2009; Wang et al., 2015; Mondal et al., 2012; Park et al., 2012; liu et al., 2015; This study |
| 278 | c.841delG | frameshift | Caucasian (German) | Opitz et al., 2004 |
| 279 | c.842delA | p.Glu281fsX37 | German | Opitz et al., 2004 |
| 280 | c.848A>T | p.Asn283Ile | in dbSNP (submit CSHL-HAPMAP) | Albinism Database |
| 281 | c.862_863delTT | frameshift | Chinese | Tsai et al., 1999 |
| 282 | c.863delT | frameshift | Korean | Spritz, 1993 |
| 283 | c.863T>C | p.Leu288Ser | Caucasian | Oetting et al., 1994a |
| 284 | c.864A>T | p.Leu288Phe | Caucasian | Hutton and Spritz, 2008b |
| 285 | c.865T>C | p.Cys289Arg | Caucasian | Oetting et al., 1998; Opitz et al., 2004 |
| 286 | c.865T>G | p.Cys289Gly | Chinese | Tsai et al., 1999 |
| 287 | c.866G>A | p.Cys289Tyr | African American | King et al., 2003 |
| 288 | c.880G>A | p.Glu294Lys | Moroccan/Sephardic Jew Caucasian | Gershoni-baruch et al., 1994; Spritz et al., 1997, King et al., 2003; Hutton and Spritz, 2008b |
| 289 | c.881A>G | p.Glu294Gly | Caucasian (German) | Passmore et al., 1999 |
| 290 | c.883G>A | p.Gly295Arg | Indian | Miyamura et al., 2005 |
| 291 | c.883G>T | p.Gly295Term | Chinese | Zheng et al., 2011 |
| 292 | c.892C>T | p.Arg298Trp | not reported | Simeonov et al., 2013 |
| 293 | c.895C>A | p.Arg299Ser | Caucasian, Taiwanese | Spritz et al., 1997; King et al., 2003; Hutton and Spritz, 2008b; Lin et al., 2006; Wang et al., 2009 |
| 294 | c.895C>T | p.Arg299Cys | Danish, Pakistani | Gronskov et al., 2009; Gul et al., 2019 |
| 295 | c.896G>A | p.Arg299His | Caucasian, Arab Christian, Korean | Tripathi et al., 1992; Gershoni-baruch et al., 1994; Park et al., 1997; King et al., 2003; Zahed et al., 2005; Hutton and Spritz, 2008b; Wang et al., 2015 |
| 296 | c.902C>T | p.Pro301Leu | Iranian | Khordadpoor-Deilamani et al., 2015 |
| 297 | c.911_914delATGA | frameshift | Caucasian | King et al., 2003 |
| 298 | c.913G>A | p.Asp305Asn | Caucasian | Opitz et al., 2004 |
| 299 | c.915C>A | p.Asp305Glu | Caucasian | King et al., 2003 |
| 300 | c.923G>C | p.Arg308Thr | in dbSNP (submit by LEE & SNP500) | Albinism Database |
| 301 | c.929_930insC | frameshift | Japanese, Korean | Tomita et al., 1989; Park et al., 1997; Goto et al., 2004; Wang et al., 2009; Park et al., 2012 |
| 302 | c.934C>G | p.Leu312Val | Hmong | Oetting et al., 1998 |
| 303 | c.937_944delCCCTCTTC | frameshift | eastern Indian (west Bengal) | Sundaresan et al., 2004 |
| 304 | c.938C>G | p.Pro313Arg | Hmong | Oetting et al., 1998 |
| 305 | c.943_948delTCAGCT | p.Ser315_Ala316del | Pakistani | Forshew et al., 2005 |
| 306 | c.953T>A | p.Val318Glu | German | Opitz et al., 2004 |
| 307 | c.962G>T | p.Cys321Phe | Not reported | Preising et al., 2011 |
| 308 | c. 969T>A | p.Ser323Arg | Not reported | Summer et al., 2014 |
| 309 | c.973A>G | p.Thr325Ala | Caucasian | Spritz et al., 1997; Hutton and Spritz, 2008b |
| 310 | c.976C>T | p.Gln326Term | southern Indian (Andhra Pradesh) | Chaki et al., 2006 |
| 311 | c.978delA | frameshift | Caucasian | Simeonov et al., 2013 |
| 312 | c.980A>G | p.Tyr327Cys | Moroccan, Caucasian | Badens et al., 2006; King et al., 2003 |
| 313 | c.982G>C | p.Glu328Gln | indo-Pakistani | Tripathi et al., 1993 |
| 314 | c.982G>A | p.Glu328Lys | Not reported | Preising et al., 2011 |
| 315 | c.985T>C | p.Ser329Pro | German | Opitz et al., 2004 |
| 316 | c.995T>C | p.Met332Thr | Caucasian | King et al., 2003; Opitz et al., 2004 |
| 317 | c.996G>A | p.Met332Ile | Danish | Gronskov et al., 2009 |
| 318 | c.1011_1012insC | p.Pro292fsX7 | Not reported | Tomita et al., 1989 |
| 319 | c.1015A>G | p.Ser339Gly | Jamaican | Oetting et al., 1999; Spritz et al., 1997 |
| 320 | c.1018T>C | p.Phe340Leu | Caucasian | Oetting et al., 1998 |
| 321 | c.1034A>G | p.Glu345Gly | German | Opitz et al., 2004 |
| 322 | c.1036G>T | p.Gly346Term | Caucasian | Oetting et al., 1994, King et al., 2003 |
| 323 | c.1036+2T>G | splice site | Caucasian | King et al., 2003 |
| 324 | c.1037-1G>A | splice site | Caucasian | Spritz et al., 1997; Hutton and Spritz, 2008a |
| 325 | c.1037-2T>A and c.1037-10_11delTT | splice site | Korean, Japanese | Park et al., 1997 |
| 326 | c.1037-7T>A and c.1037-10_11delTT | splice site | Japanese | Goto et al., 2004; wang et al., 2015 |
| 327 | c.1037-7T>A | splice site | Caucasian, Moroccan/Sephardic Jew, Japanese, European | Spritz, 1993; Gershoni-baruch et al., 1994; King et al., 2003; Hutton and Spritz, 2008a, Hutton and Spritz, 2008b; Gul et al., 2019 |
| 328 | c.1037G>A | p.Gly346Glu | Caucasian, Lebanese | Oetting et al., 1994a; Zahed et al., 2005 |
| 329 | c.1037G>T | p.Gly346Val | Indian | Miyamura et al., 2005 |
| 330 | c.1063G>C | p.Ala355Pro | Caucasian | Spritz, 1993; Opitz et al., 2004 |
| 331 | c.1064C>A | p.Ala355Glu | Caucasian | Oetting et al., 1994a |
| 332 | c.1064C>T | p.Ala355Val | Caucasian | Simeonov et al., 2013 |
| 333 | c.1065delG | frameshift | Indian | Mondal et al., 2012 |
| 334 | c.1075C>T | p.Gln359Term | Caucasian, German | Spritz et al., 1997; Opitz et al., 2004; Hutton and Spritz, 2008b |
| 335 | c.1076A>T | p.Gln359Leu | Lebanese | Zahed et al., 2005 |
| 336 | c.1078A>G | p.Ser360Gly | Lebanese | Zahed et al., 2005 |
| 337 | c.1083C>A | p.Ser361Arg | Caucasian | Summers, 1996 |
| 338 | c.1087C>T | p.His363Tyr | German | Opitz et al., 2004 |
| 339 | c.1090A>C | p.Asn364His | not reported | Simeonov et al., 2013 |
| 340 | c.1092T>G | p.Asn364Lys | Chinese | Lin et al., 2014 |
| 341 | c.1099C>T | p.His367Tyr | British | Breimer et al., 1994 |
| 342 | c.1100A>G | p.His367Arg | Caucasian | Oetting and King, 1994 |
| 343 | c.1106A>G | p.Tyr369Cys | Caucasian | King et al., 2003 |
| 344 | c.1110G>A | p.Met370Ile | Not reported | Preising et al., 2011 |
| 345 | c.1109T>C | p.Met370Thr | British | Breimer et al., 1994 |
| 346 | c. 1108A>G | p.Met370Val | Not availabe | Summer et al., 2014 |
| 347 | c.1111A>T | p.Asn371Tyr | Caucasian (German) | Passmore et al., 1999 |
| 348 | c.1112A>C | p.Asn371Thr | Caucasian | Oetting et al., 1991b; King et al., 2003, Oetting and king, 1994 |
| 349 | c.1111A>G | p.Asn371Asp | Spanish | [Vidal-Ríos](https://pubmed.ncbi.nlm.nih.gov/?term=Vidal-R%C3%ADos+P&cauthor_id=23085315) et al., 2013 |
| 350 | c.1114G>A | p.Gly372Arg | eastern Indian (west Bengal) | Chaki et al., 2006 |
| 351 | c.1114delG | (p. G372fsX112) * | Chinese | Wang et al., 2015 |
| 352 | c.1115+ 2 T > C | IVS3 + 2 T > C | Chinese | Liu et al., 2014 |
| 353 | c.1118C>A | p.Thr373Lys | Caucasian, Libyan Jew, European, German | Oetting et al., 1991a; Oetting and king, 1994; Gershoni-baruch et al., 1994; King et al., 2003; Opitz et al., 2004; Hutton and Spritz, 2008a; Hutton and Spritz, 2008b |
| 354 | c.1126C>T | p.Gln376Term | indo Pakistani | Tripathi et al., 1993 |
| 355 | c.1130T>C | p.Val377Ala | Not reported | Preising et al., 2011 |
| 356 | c.1132C>T | p.Gln378Term | Caucasian | King and Oetting, 1992; Hutton and Spritz, 2008b |
| 357 | c.1132C>A | p.Gln378Lys | German | Opitz et al., 2004 |
| 358 | c.1138T>C | p.Ser380Pro | Caucasian | Spritz, 1993; Hutton and Spritz, 2008b |
| 359 | c.1138_1158del | frameshift | Caucasian | Simeonov et al., 2013 |
| 360 | c.1141G>A | p.Ala381Thr | Not reported | Patrosso et al., 2008 |
| 361 | c.1146C>G | p.Asn382Lys | Chinese | Wei et al., 2010 |
| 362 | c.1146C>A | p.Asn382Lys | Caucasian | Oetting et al., 1991b; Oetting and king, 1994; Hutton and Spritz, 2008b |
| 363 | c.1147G>A | p.Asp383Asn | Caucasian, Korean | Park et al., 1997; King et al., 2003; Opitz et al., 2004; Hutton and Spritz, 2008a; Hutton and Spritz, 2080b |
| 364 | c.1150C>G | p.Pro384Ala | not reported | Simeonov et al., 2013 |
| 365 | c.1164delT | frameshift | Caucasian, German | Tripathi et al., 1992; Opitz et al., 2004; Hutton and Spritz, 2008b |
| 366 | c.1167_1168insT | frameshift | German | Opitz et al., 2004 |
| 367 | c.1168C>G | p.His390Asp | Caucasian | Spritz, 1993; Hutton and Spritz, 2008b |
| 368 | c.1172C>A | p.Ala391Glu | Caucasian | King et al., 2003 |
| 369 | c.1176delT | frameshift | Caucasian | King et al., 2003 |
| 370 | c.1177G>T | p.Val393Phe | Caucasian | Oetting et al., 1994a; Opitz et al., 2004 |
| 371 | c.1177delG | p.Val393fs | Italian | Gargiulo et al., 2011 |
| 372 | c. 1178T>A | p.Val393Asp | Not available | Summer et al., 2014 |
| 373 | c.1180delG | p.Asp394fsX90 | German | Opitz et al., 2004 |
| 374 | c.1183A>C | p.Ser395Arg | German | Opitz et al., 2004 |
| 375 | c.1184G>A | p.Ser395Asn | Caucasian | Spritz et al., 1997; Hutton and Spritz, 2008b |
| 376 | c.1184+1G>A | splice site mutation | not reported | Simeonov et al., 2013 |
| 377 | c.1184+3T>G | splice site | German | Opitz et al., 2004 |
| 378 | c.1185-1G>T | splice site | Moroccan | Rooryck et al., 2008 |
| 379 | c.1193A>G | p.Glu398Gly | Japanese | Miyamura et al., 2005 |
| 380 | c.1193A>T | p.Glu398Val | German | Opitz et al., 2004 |
| 381 | c.1193A>C | p.Glu398Ala | German | Opitz et al., 2004 |
| 382 | c.1195C>T | p.Gln399Term | Chinese | Wu et al., 2012 |
| 383 | c.1196delA | frameshift | Chinese | Wei et al., 2011 |
| 384 | c.1198T>G | p.Trp400Gly | Chinese | Sun et al., 2018 |
| 385 | c.1199G>T | p.Trp400Leu | Chinese | Tsai et al., 1999; Lin et al., 2006; Wang et al., 2009 |
| 386 | c.1200G>T | p.Trp400Cys | French | Rooryck et al., 2008 |
| 387 | c.1284G>A | p.Arg402Gln | Caucasian | Fukai et al., 1995 |
| 388 | c.1204C>G | p.Arg402Gly | Caucasian | Oetting et al., 1994a |
| 389 | c.1204C>T | p.Arg402Term | Caucasian, Lebanese | Spritz, 1993; Gershoni-baruch et al.,1994; King et al., 2003; Opitz et al., 2004; Zahed et al., 2005; Hutton and Spritz, 2008a; Hutton and Spritz, 2008b |
| 390 | c.1205G>T | p.Arg402Leu | German | Opitz et al., 2004 |
| 391 | c.1207delA | frameshift | Lebanese | Zahed et al., 2005 |
| 392 | c.1209G>C | p.Arg403Ser | German | Opitz et al., 2004 |
| 393 | c.1209G>T | p.Arg403Ser | Caucasian, German | Tripathi et al., 1992; Opitz et al., 2004; Hutton and Spritz, 2008b |
| 394 | c.1210C>A | p.His404Asn | German | Opitz et al., 2004 |
| 395 | c.1211A>C | p.His404Pro | Caucasian | Oetting et al., 1998 |
| 396 | c.1214G>T | p.Arg405Leu | German | Opitz et al., 2004 |
| 397 | c.1217C>T | p.Pro406Leu | Caucasian | Giebel et al., 1991c; King et al., 2003; Opitz et al., 2004; Hutton and Spritz, 2008b, Wei et al., 2010 |
| 398 | c.1224A>C | p.Gln408His | German | Opitz et al., 2004 |
| 399 | c.1227A>C | p.Glu409Asp | German | Opitz et al., 2004 |
| 400 | c.1231T>C | p.Tyr411His | Pakistani | Jaworek et al., 2012 |
| 401 | c.1234C>G | p.Pro412Ala | Caucasian | King et al., 2003 |
| 402 | c.1237G>T | p.Glu413Term | German | Opitz et al., 2004 |
| 403 | c.1246G>T | p.Ala416Ser | German | Opitz et al., 2004 |
| 404 | c.1250C>A | p.Pro417His | German | Opitz et al., 2004 |
| 405 | c.1255G>A | p.Gly419Arg | Caucasian, indo-Pakistani | King et al., 1991b; Tripathi et al., 1993; King et al., 2003; Optiz et al., 2004; Chaki et al., 2006, Hutton and Spritz, 2008b, Gul et al., 2017; Gul et al., 2019 |
| 406 | c.1262+ 1delG | IVS4 + 1delG | Chinese | Liu et al., 2014 |
| 407 | c.1264C>T | p.Arg422Trp | Caucasian | King et al., 2003 |
| 408 | c.1265G>A | p.Arg422Gln | Caucasian | Giebel et al., 1991b; Opitz et al., 2004; Hutton and Spritz, 2008 |
| 409 | c.1271C>T | p.Ser424Phe | German | Opitz et al., 2004 |
| 410 | c.del1276–82 | Frameshift | Iranian | Khordadpoor-Deilamani et al., 2016 |
| 411 | c.1277T>A | p.Met426Lys | German | Opitz et al., 2004 |
| 412 | c.1279G>T | p.Val427Phe | Danish | Gronskov et al., 2009 |
| 413 | c.1280T>G | p.Val427Gly | German | Opitz et al., 2004 |
| 414 | c.1291C>A | p.Pro431Thr | indo-Pakistani, northern European | Spritz, 1993; King et al., 2003 |
| 415 | c.1292C>T | p.Pro431Leu | indo-Pakistani, Japanese | Tripathi et al., 1993; Zahed et al., 2005 |
| 416 | c.1298A>G | p.Tyr433Cys | Lebanese | Zahed et al., 2005 |
| 417 | c.1299C>G | p.Tyr433Ter | Indian | Chaki et al., 2011 |
| 418 | c.1301G>T | p.Arg434Ile | German | Opitz et al., 2004 |
| 419 | c.1303A>G | p.Asn435Asp | German | Opitz et al., 2004 |
| 420 | c.1306G>C | p.Gly436Arg | European | King et al., 2003 |
| 421 | c.1309G>A | p.Asp437Asn | not reported | Simeonov et al., 2013 |
| 422 | c.1314_1317delCTTT | frameshift | Caucasian | King and Oetting, 1992 |
| 423 | c.1315T>G | p.Phe439Val | Caucasian | Passmore et al., 1999 |
| 424 | c.1315_1317TTT>C | frameshift | not reported | Breimer et al., 1995 |
| 425 | c.1322delC | p.Ser441Tyrfs*44 | Arabian | Khan et al., 2016 |
| 426 | c.1324T>C | p.Ser442Pro | not reported | Preising et al., 2011 |
| 427 | c.1331A>G | p.Asp444Gly | German | Opitz et al., 2004 |
| 428 | c.1336G>A | p.Gly446Ser | Caucasian | Tripathi et al., 1992; King et al., 2003; Hutton and Spritz, 2008b |
| 429 | c.1337G>A | p.Gly446Val | Chinese | Liu et al., 2014 |
| 430 | c.1342G>A | p.Asp448Asn | Caucasian, Lebanese | Tripathi et al., 1992; King et al., 2003; Opitz et al., 2004; Zahed et al., 2005; Hutton and Spritz, 2008b |
| 431 | c.1346A>G | p.Tyr449Cys | Puerto Rican | King et al., 2003 |
| 432 | c.1349_1350insGG | frameshift | Chinese | Wang et al., 2009 |
| 433 | c.1352A>G | p.Tyr451Cys | Italian | Gargiulo et al., 2011 |
| 434 | c.1354C>G | p.Leu452Val | Danish | Gronskov et al., 2009 |
| 435 | c.1357C>T | p.Gln453Term | Pakistani | King and Oetting, 1992 |
| 436 | c.1366+1G>A | splice mutation | French | Rooryck et al., 2008 |
| 437 | c.1366+3A>T | splice mutation | Asian | Rooryck et al., 2008 |
| 438 | c.1366+4A>G | splice mutation | Caucasian | Spritz, 1993; Hutton and Spritz, 2008b |
| 439 | c.1366+5A>G | splice mutation | Caucasian | Spritz et al., 1997 |
| 440 | c.1379T>C | p.Phe460Ser | in dbSNP (submit by TOMITA) | Albinism Database |
| 441 | c.1379_1380delTT | frameshift | eastern Indian | Sundaresan et al., 2004; Chaki et al., 2006 |
| 442 | c.1393_1394insT | p.Lys465Term | French | Rooryck et al., 2008 |
| 443 | c.1423_1433del11 | frameshift | Caucasian | King et al., 2003 |
| 444 | c.1424G > A | p.Trp475Term | Pakistani | Gul et al., 2019 |
| 445 | c.1425G>A | p.Trp475Term | Chinese | Wang et al., 2009 |
| 446 | c.1442C>A | p.Ala481Glu | Chinese | Wei et al., 2010 |
| 447 | c.1467_1468insT | frameshift | Caucasian | Chintamaneni et al., 1991; King and Oetting, 1992, King et al., 2003; Opitz et al., 2004; Hutton and Spritz, 2008a, Hutton and Spritz, 2008b |
| 448 | c.1469C>A | p.Ala490Asp | Caucasian | Simeonov et al., 2013 |
| 449 | c.1469C>G | p.Ala490Gly | Caucasian | King et al., 2003 |
| 450 | c.1501_1502insC | p.R501fsX8 | Caucasian | Giebel et al., 1991c; Opitz et al., 2004 |
| 451 | c.1516C>T | p.Gln506Term | Chinese | Wei et al., 2010 |
| 452 | c.1185-?_1367+?del | NA | southern Indian | Ray et al., 2005; Chaki et al., 2006 |
| 453 | c.(?_-30)_(*220_?) | NA | Ashkenazi, French | Schnur et al., 1996, Coupry et al., 2001; Rooryck et al., 2008; Hutton and Spritz, 2008b |
| 454 | del exon 2 | 8.03 kb | Not reported | [Morice-Picard et al., 2014](http://www.ncbi.nlm.nih.gov/sites/entrez?cmd=Retrieve&db=PubMed&list_uids=24118800&dopt=Abstract) |
| 455 | del TYR exon 3–5, | 340.5 kb | Not reported | Morice-Picard et al., 2014 |

**Table S2: Known *SLC45A2* Mutations reported to date**

| **No** | **cDNA** | **Protein** | **Ethnicities** | **References** |
| --- | --- | --- | --- | --- |
| 1 | c.113A>G | p.His38Arg | Moroccan | Konno et al., 2009 |
| 2 | c.125T > C | p.Met42Thr | Japanese | Okamura et al., 2016 |
| 3 | c.126G>A | p.Met42Ile | Indian | Sengupta et al., 2007 |
| 4 | c.126G>T | p.Met42Ile | Italian | Gargiulo et al., 2011 |
| 5 | c.130G>A | p.Gly44Arg | Caucasian | Hutton and Spritz, 2008b |
| 6 | c.143_145delGCT | p.C48del | Chinese | Wei et al., 2011 |
| 7 | c.146A>G | p.Tyr49Cys | Japanese | Inagaki et al., 2006 |
| 8 | c.149C > T | p.Ala50Val | Japanese | Okamura et al., 2016 |
| 9 | c.157G > C | p.Ala53Pro | Japanese | Okamura et al., 2016 |
| 10 | c.152_153delTG | frameshift | Chinese | Wei et al., 2011 |
| 11 | c.150_160insGGTGGAGGCAG | frameshift | Italian | Gargiulo et al., 2011 |
| 12 | c.168_173delGACCCC | p.T57_P58del | Chinese | Wei et al., 2011 |
| 13 | c.170C > T | p.Thr57Ile | Japanese | Okamura et al., 2016 |
| 14 | c.172C>G | p.Pro58Ala | German | Rundshagen et al., 2004 |
| 15 | c.172C>T | p.Pro58Ser | Japanese | Inagaki et al., 2004 |
| 16 | c.179T>G | p.Leu60Arg | Caucasian | Simeonov et al., 2013 |
| 17 | c.190G>A | p.Gly64Ser | Indian | Sengupta et al., 2007 |
| 18 | c.192delT | p.Gly64fsX112 | Japanese | [Okamura](http://www.ncbi.nlm.nih.gov/sites/entrez?cmd=Retrieve&db=PubMed&list_uids=24461674&dopt=Abstract)  et al., 2014 |
| 19 | c.210C*>*A | p.Tyr70Term | Japanese | Lin et al., 2014 |
| 20 | c.217G > T | p.Val73Leu | Japanese | Okamura et al., 2016 |
| 21 | c.233C > T | p.Pro78Leu | Japanese | Okamura et al., 2014 |
| 22 | c.251T>C | p.Leu84Pro | Pakistani | Kausar et al 2013 |
| 23 | c.264delC | p.F88fsX23 | central Anatolia | Ikinciogullari et al., 2005 |
| 24 | c.265G>A | p.Gly89Arg | Japanese | Inagaki et al., 2006 |
| 25 | c.269_274CGGCCA>GC | frameshift | Japanese | Inagaki et al., 2004 |
| 26 | c.277G>A | p.Asp93Asn | Korean | Rooryck et al., 2008 |
| 27 | c.280CNG | p.His94Asp | Not reported | Mauri et al., 2014 |
| 28 | c.298G>A | p.Gly100Ser | Caucasian | Hutton and Spritz, 2008b |
| 29 | c.301C>T | p.Arg101Cys | Caucasian | Hutton and Spritz, 2008b |
| 30 | c.328G>A | p.Gly110Arg | Chinese | Wei et al., 2010 |
| 31 | c.375T>A | p.Val126Asp | Italian | Gargiulo et al., 2011 |
| 32 | c.386-1G>A | IVS1-1G>A | Turkish | Newton et al., 2001 |
| 33 | c.426_429dupAAGT | frameshift | Japanese | Inagaki et al., 2004 |
| 34 | c.452T>C | p.Leu151Pro | Chinese | Wei et al., 2010 |
| 35 | c.459_470del  TTTTGCTGCCGA | p.Ala155_Phe158del | Chinese | Qiu et al., 2018 |
| 36 | c.463delG | frameshift | Chinese | Wei et al., 2010 |
| 37 | c.469G>A | p.Asp157Asn | Japanese, Korean | Inagaki et al., 2004; Sengupta et al., 2007; Ko et al., 2012; Park et al., 2012; Okamura et al ., 2014 |
| 38 | c.478G>C | p.Asp160His | Chinese, Japanese | Li et al., 2008; [Lin et al., 2014](http://www.ncbi.nlm.nih.gov/sites/entrez?cmd=Retrieve&db=PubMed&list_uids=24721949&dopt=Abstract) |
| 39 | c.551C>T | p.Ala184Val | Chinese | Wei et al., 2011 |
| 40 | c.562-1118_885+ 4923del | NA | Chinese | Xu et al., 2012 |
| 41 | c.563G>T | p.Gly188Val | Japanese | Inagaki et al., 2004 |
| 42 | c.673 G>T | p.Gly188Asp | Chinese | Wei et al., 2013 |
| 43 | c.593G>A | p.Gly198Asp | Caucasian | Hutton and Spritz, 2008b |
| 44 | c.606G>C | p.Trp202Cys | German | Rundshagen et al., 2004 |
| 45 | c.662_664delTCT | p.F221del | German | Rundshagen et al., 2004 |
| 46 | c.663_665delCTC | p.L222del | Chinese | Wei et al., 2011 |
| 47 | c.686G>A | p.Cys229Tyr | Japanese, Korean | Inagaki et al., 2006, Park et al., 2012 |
| 48 | c.699T>A | p.His233Gln | Chinese | Wei et al., 2010 |
| 49 | c.739A>T | p.Lys247Term | Moroccan | Rooryck et al., 2008 |
| 50 | c.793 A>T | p.Met265Leu | Not reported | Mauri et al., 2014 |
| 51 | c.798C>G | p.Tyr266Term | Chinese | Wei et al., 2010 |
| 52 | c.817_818insGA | frameshift | Caucasian | Simeonov et al 2013 |
| 53 | c.834C>G | p.Tyr278Term | Caucasian | Hutton and Spritz, 2008b |
| 54 | c.870delC | p.N290KfsX11 | Chinese | Pang et al., 2011 |
| 55 | c.889-6T>G | Splice mutation | Pakistani | Kausar et al., 2013 |
| 56 | c.889-?_1032+?del | exon 4 deletion | French | Rooryck et al., 2008 |
| 57 | c.892C>T | p.Arg298Cys | Caucasian | Hutton and Spritz, 2008 |
| 58 | c.904A>G | p.Thr302Ser | Indian | Sengupta et al., 2007 |
| 59 | c.950A>G | p.Tyr317Cys | German | Rundshagen et al., 2004 |
| 60 | c.974T>C | p.Leu325Pro | Indian | Sengupta et al., 2007 |
| 61 | c.986delC | p.Thr329fsX68 | German | Rundshagen et al., 2004; Hutton and Spritz, 2008b, Morice-Picard et al ., 2014 |
| 62 | c.1004T>G | p.Met335Arg | Caucasian | Hutton and Spritz, 2008 |
| 63 | c.1030C > T | p.Gln344Term | Japanese | Okamura et al ., 2016 |
| 64 | c.1032C>A | p.Gln344His | Not reported | Morice-Picard et al., 2014 |
| 65 | c.1033-2A>T | splice site | Chinese | Wei et al., 2011 |
| 66 | c.1042C>T | p.Arg348Cys | Indian | Sengupta et al., 2007 |
| 67 | c.1045G>A | p.Gly349Arg | Chinese | Wei et al., 2009 |
| 68 | c.1076_1077delAG | frameshift | Caucasian | Hutton and Spritz, 2008a |
| 69 | c.1082T>C | p.Leu361Pro | German | Rundshagen et al., 2004 |
| 70 | c.1102G>A | p.Glu368Lys | Chinese | Wei et al., 2010 |
| 71 | c.1108G>C | p.Gly370Arg | Italian | Gargiulo et al., 2011 |
| 72 | c.1121delT | p.L374fsX397 | Brazilian | Lezirovitz et al., 2006 |
| 73 | c.1156+1G>A | Splice site | Not reported | Mauri et al., 2014 |
| 74 | c.1156+2dupT | IVS5+2dupT | Italian | Straniero et al., 2015 |
| 75 | c.1156+3_1156+6delAAGT | splice site | Chinese | Li et al., 2008 |
| 76 | c.1166_1167delAA | frameshift | Caucasian | Hutton and Spritz, 2008a |
| 77 | c.1179_1203dup | p.Y401X | German | Rundshagen et al., 2004 |
| 78 | c.1210G>A | p.Gly404Arg | Chinese | Wei et al., 2011 |
| 79 | c.1256C>T | p.Pro419Leu | Chinese | Wei et al., 2010 |
| 80 | c.1273delC | p.Leu425Trpfs | Iranian | Khordadpoor-Deilamani et al., 2015 |
| 81 | c.1280T>C | p.Leu427Pro | Italian | Gargiulo et al., 2011 |
| 82 | c.1304C>A | p.Ser435Tyr | Chinese | Wei et al., 2011 |
| 83 | c.1309A>G | p.Thr437Ala | Japanese | Inagaki et al., 2006 |
| 84 | c.1318A>G | p.Thr440Ala | Japanese | Inagaki et al., 2006 |
| 85 | c.1331_1332insA | p.Asn444LysfsX5 | Pakistani | This study |
| 86 | c.1405delG | frameshift | Japanese | Inagaki et al., 2004 |
| 87 | c.1418G>A | p.Gly473Asp | Japanese | Inagaki et al., 2006 |
| 88 | c.1429G>A | p.Ala477Thr | German | Rundshagen et al., 2004 |
| 89 | c.1457C>T | p.Ala486Val | German | Rundshagen et al., 2004 |
| 90 | c.1502C>A | p.Ala501Asp | Caucasian | Hutton and Spritz, 2008a |
| 91 | c.1519G>C | p.Val507Leu | Japanese | Inagaki et al., 2004 |
| 92 | c.1532C>A | p.Ala511Glu | Italian | Gargiulo et al., 2011 |
| 93 | c.1532C>T | p.Ala511Val | Pakistani | Kausar et al., 2013; Gul et al., 2019 |
| 94 | c.1567_1574dupGCTCTCTT | p.F525fsX15 | German | Rundshagen et al., 2004 |
| 95 | Homo del *SLC45A2* | na | Moroccan | Verhagen et al., 2012 |
| 96 | del SLC45A2 AMACR C1QTNF3 | 79.87 kb | Not reported | Morice-Picard et al., 2014 |

**References for supplementary tables**

1. Albinism Database, University of Minnesota. (Accessed June13, 2020, at <http://albinismdb.med.umn.edu/>.)
2. Aquaron R, Hesse S, Badens C, et al. [New nonsense mutation (p.E250X) in the tyrosinase gene of a patient with oculocutaneous albinism type 1A]. Ann Dermatol Venereol 2009;136:57-59.
3. Badens C, Courrier S, Aquaron R. A novel mutation (delAACT) in the tyrosinase gene in a Cameroonian black with type 1A oculocutaneous albinism. J Dermatol Sci 2006;42:121-124.
4. Breimer LH, Winder AF, Jay B, et al. Initiation codon mutation of the tyrosinase gene as a cause of human albinism. Clin Chim Acta 1994;227:17-22.
5. Breimer LH, Winder AF, Panayiotidis P, et al. A trinucleotide deletion together with a base duplication event at codon 439 in the human tyrosinase gene identifies a mutational hotspot. Clin Chim Acta 1995;243:35-42.
6. Camand O, Marchant D, Boutboul S, et al. Mutation analysis of the tyrosinase gene in oculocutaneous albinism. Hum Mutat 2001;17:352.
7. Chaki M, Sengupta M, Mukhopadhyay A, et al. OCA1 in different ethnic groups of india is primarily due to founder mutations in the tyrosinase gene. Ann Hum Genet 2006;70:623-630.
8. Chintamaneni CD, Halaban R, Kobayashi Y, et al. A single base insertion in the putative transmembrane domain of the tyrosinase gene as a cause for tyrosinase-negative oculocutaneous albinism. Proc Natl Acad Sci U S A 1991;88:5272-5276.
9. Chong JX, Ouwenga R, Anderson RL, et al. A population-based study of autosomal-recessive disease-causing mutations in a founder population. *Am J Hum Genet*. 2012; 91(4):608-620.
10. Coupry I, Taine L, Goizet C, et al. Leucodystrophy and oculocutaneous albinism in a child with an 11q14 deletion. J Med Genet 2001;38:35-38.
11. Forshew T, Khaliq S, Tee L, et al. Identification of novel TYR and TYRP1 mutations in oculocutaneous albinism. Clin Genet 2005;68:182-184.
12. Fukai K, Holmes SA, Lucchese NJ, et al. Autosomal recessive ocular albinism associated with a functionally significant tyrosinase gene polymorphism. *Nat Genet*. 1995;9(1):92-95.
13. Gargiulo A, Testa F, Rossi S, et al. Molecular and clinical characterization of albinism in a large cohort of Italian patients. Invest Ophthalmol Vis Sci 2011;52:1281-1289.
14. Giebel LB, Musarella MA, Spritz RA. A nonsense mutation in the tyrosinase gene of Afghan patients with tyrosinase negative (type IA) oculocutaneous albinism. J Med Genet 1991a;28:464-467.
15. Giebel LB, Strunk KM, King RA, et al. A frequent tyrosinase gene mutation in classic, tyrosinase-negative (type IA) oculocutaneous albinism. Proc Natl Acad Sci U S A 1990;87:3255-3258.
16. Giebel LB, Tripathi RK, King RA, et al. A tyrosinase gene missense mutation in temperature-sensitive type I oculocutaneous albinism. A human homologue to the Siamese cat and the Himalayan mouse. J Clin Invest 1991b;87:1119-1122.
17. Giebel LB, Tripathi RK, Strunk KM, et al. Tyrosinase gene mutations associated with type IB ("yellow") oculocutaneous albinism. Am J Hum Genet 1991c;48:1159-1167.
18. Gronskov K, Ek J, Sand A, et al. Birth prevalence and mutation spectrum in danish patients with autosomal recessive albinism. Invest Ophthalmol Vis Sci 2009;50:1058-1064.
19. Gershoni-Baruch R, Rosenmann A, Droetto S, et al. Mutations of the tyrosinase gene in patients with oculocutaneous albinism from various ethnic groups in Israel. Am J Hum Genet 1994;54:586-594.
20. Ghodsinejad Kalahroudi V, Kamalidehghan B, Arasteh Kani A, et al. Two novel tyrosinase (TYR) gene mutations with pathogenic impact on oculocutaneous albinism type 1 (OCA1). *PLoS One*. 2014;9(9):e106656.
21. Goto M, Sato-Matsumura KC, Sawamura D, et al. Tyrosinase gene analysis in Japanese patients with oculocutaneous albinism. J Dermatol Sci 2004;35:215-220.
22. Gul H, Ali MZ, Khan E, et al. Ophthalmo-genetic analysis of Pakistani patients with nonsyndromic oculocutaneous albinism through whole exome sequencing. *J Pak Med Assoc*. 2017;67(5):790-792
23. Gul H, Shah AH, Harripaul R, et al. Genetic studies of multiple consanguineous Pakistani families segregating oculocutaneous albinism identified novel and reported mutations. *Ann Hum Genet*. 2019;83(4):278-284
24. Human gene mutation database. (Accessed June 13,2020, at <http://www.hgmd.cf.ac.uk/ac/>.)
25. Hutton SM, Spritz RA. A comprehensive genetic study of autosomal recessive ocular albinism in Caucasian patients. Invest Ophthalmol Vis Sci 2008a;49:868-872.
26. Hutton SM, Spritz RA. Comprehensive analysis of oculocutaneous albinism among non-Hispanic caucasians shows that OCA1 is the most prevalent OCA type. J Invest Dermatol 2008b;128:2442-2450.
27. Ikinciogullari A, Tekin M, Dogu F, et al. Meningococccal meningitis and complement component 6 deficiency associated with oculocutaneous albinism. Eur J Pediatr 2005;164:177-179.
28. Inagaki K, Suzuki T, Ito S, et al. Oculocutaneous albinism type 4: six novel mutations in the membrane-associated transporter protein gene and their phenotypes. Pigment Cell Res 2006;19:451-453.
29. Inagaki K, Suzuki T, Shimizu H, et al. Oculocutaneous albinism type 4 is one of the most common types of albinism in Japan. Am J Hum Genet 2004;74:466-471.
30. Ito S, Suzuki T, Inagaki K, et al. Two novel mutations detected in Japanese patients with oculocutaneous albinism. J Dermatol Sci 2006;44:116-118
31. Jaworek TJ, Kausar T, Bell SM, et al. Molecular genetic studies and delineation of the oculocutaneous albinism phenotype in the Pakistani population. Orphanet J Rare Dis 2012;7:44.
32. Kausar T, Jaworek TJ, Tariq N, et al. Genetic studies of TYRP1 and SLC45A2 in Pakistani patients with nonsyndromic oculocutaneous albinism. *J Invest Dermatol*. 2013;133(4):1099-1102.
33. Khan AO, Tamimi M, Lenzner S, et al. Hermansky-Pudlak syndrome genes are frequently mutated in patients with albinism from the Arabian Peninsula. *Clin Genet*. 2016;90(1):96-98.
34. Khordadpoor-Deilamani F, Akbari MT, Karimipoor M, et al. Sequence analysis of tyrosinase gene in ocular and oculocutaneous albinism patients: introducing three novel mutations. *Mol Vis*. 2015;21:730-735.
35. Khordadpoor-Deilamani F, Akbari MT, Karimipoor M, et al. Homozygosity mapping in albinism patients using a novel panel of 13 STR markers inside the nonsyndromic OCA genes: introducing 5 novel mutations. *J Hum Genet*. 2016;61(5):373-379.
36. King RA, Pietsch J, Fryer JP, et al. Tyrosinase gene mutations in oculocutaneous albinism 1 (OCA1): definition of the phenotype. Hum Genet 2003;113:502-513.
37. King RA, Mentink MM, Oetting WS. Non-random distribution of missense mutations within the human tyrosinase gene in type I (tyrosinase-related) oculocutaneous albinism. Mol Biol Med 1991a;8:19-29.
38. King RA, Oetting WS. Molecular basis of type IA (tyrosinase negative) oculocutaneous albinism. Pigment Cell Res 1992; Suppl 2:249-253.
39. King RA, Townsend D, Oetting W, et al. Temperature-sensitive tyrosinase associated with peripheral pigmentation in oculocutaneous albinism. J Clin Invest 1991b;87:1046-1053.
40. Ko JM, Yang JA, Jeong SY, et al. Mutation spectrum of the TYR and SLC45A2 genes in patients with oculocutaneous albinism. Mol Med Report 2012;5:943-948.
41. Konno T, Abe Y, Kawaguchi M, et al. Oculocutaneous albinism type IV: A boy of Moroccan descent with a novel mutation in SLC45A2. Am J Med Genet A 2009;149A:1773-1776.
42. Lezirovitz K, Nicastro FS, Pardono E, et al. Is autosomal recessive deafness associated with oculocutaneous albinism a "coincidence syndrome"? J Hum Genet 2006;51:716-720.
43. Li H, Meng S, Zheng H, et al. A Chinese case of oculocutaneous albinism type 4 with two novel mutations. Int J Dermatol 2008;47:1198-1201.
44. Lin SY, Chien SC, Su YN, et al. Rapid genetic analysis of oculocutaneous albinism (OCA1) using denaturing high performance liquid chromatography (DHPLC) system. *Prenat Diagn*. 2006;26(5):466-470.
45. Lin YY, Wei AH, Zhou ZY, et al. A novel missense mutation of the TYR gene in a pedigree with oculocutaneous albinism type 1 from China. Chin Med J (Engl) 2011;124:3358-3361.
46. Lin YY, Wei AH, He X, et al. A comprehensive study of oculocutaneous albinism type 1 reveals three previously unidentified alleles on the TYR gene. *Eur J Dermatol*. 2014;24(2):168-173.
47. Liu J, Choy KW, Chan LW, et al. Tyrosinase gene (TYR) mutations in Chinese patients with oculocutaneous albinism type 1. Clin Experiment Ophthalmol 2010;38:37-42.
48. Liu N, Kong XD, Shi HR, et al. Tyrosinase gene mutations in the Chinese Han population with OCA1.Genet. Res.Camb. 2014 96, e14.
49. Liu Y, Wei X, Kong X, et al. Targeted Next-Generation Sequencing for Clinical Diagnosis of 561 Mendelian Diseases. PLoS One. 2015;10(9):e0139258
50. Mauri L, Barone L, Al Oum M, et al. SLC45A2 mutation frequency in Oculocutaneous Albinism Italian patients doesn't differ from other European studies. *Gene*. 2014;533(1):398-402.
51. Miyamura Y, Verma IC, Saxena R, et al. Five novel mutations in tyrosinase gene of Japanese and Indian patients with oculocutaneous albinism type I (OCA1). J Invest Dermatol 2005;125:397-398.
52. Mondal M, Sengupta M, Samanta S, et al. Molecular basis of albinism in India: evaluation of seven potential candidate genes and some new findings. *Gene*. 2012;511(2):470-474.
53. Morice-Picard F, Lasseaux E, Cailley D, et al. High-resolution array-CGH in patients with oculocutaneous albinism identifies new deletions of the TYR, OCA2, and SLC45A2 genes and a complex rearrangement of the OCA2 gene. *Pigment Cell Melanoma Res*. 2014;27(1):59-71.
54. Nakamura E, Miyamura Y, Matsunaga J, et al. A novel mutation of the tyrosinase gene causing oculocutaneous albinism type 1 (OCA1). J Dermatol Sci 2002;28:102-105.
55. Newton JM, Cohen-Barak O, Hagiwara N, et al. Mutations in the human orthologue of the mouse underwhite gene (uw) underlie a new form of oculocutaneous albinism, OCA4. Am J Hum Genet 2001;69:981-988.
56. Oetting WS, Fryer JP, King RA. Mutations of the human tyrosinase gene associated with tyrosinase related oculocutaneous albinism (OCA1). Mutations in brief no. 204. Online. Hum Mutat 1998;12:433-434.
57. Oetting WS, Fryer JP, Oofuji Y, et al. Analysis of tyrosinase gene mutations using direct automated infrared fluorescence DNA sequencing of amplified exons. Electrophoresis 1994a;15:159-164.
58. Oetting WS, Handoko HY, Mentink MM, et al. Molecular analysis of an extended family with type IA (tyrosinase-negative) oculocutaneous albinism. J Invest Dermatol 1991a;97:15-19.
59. Oetting WS, King RA. Molecular basis of albinism: mutations and polymorphisms of pigmentation genes associated with albinism. Hum Mutat 1999;13:99-115.
60. Oetting WS, King RA. Molecular basis of oculocutaneous albinism. J Invest Dermatol 1994b;103:131S-6S.
61. Oetting WS, Mentink MM, Summers CG, et al. Three different frameshift mutations of the tyrosinase gene in type IA oculocutaneous albinism. Am J Hum Genet 1991b;49:199-206.
62. Oetting WS, Witkop CJ Jr, Brown SA, et al. A frequent tyrosinase gene mutation associated with type I-A (tyrosinase-negative) oculocutaneous albinism in Puerto Rico. Am J Hum Genet 1993;52:17-23.
63. Okamura K, Araki Y, Abe Y, et al. Genetic analyses of oculocutaneous albinism types 2 and 4 with eight novel mutations. *J Dermatol Sci*. 2016;81(2):140-142.
64. Okamura K, Yoshizawa J, Abe Y, et al. Oculocutaneous albinism (OCA) in Japanese patients: five novel mutations. *J Dermatol Sci*. 2014;74(2):173-174.
65. Opitz S, Kasmann-Kellner B, Kaufmann M, et al. Detection of 53 novel DNA variations within the tyrosinase gene and accumulation of mutations in 17 patients with albinism. Hum Mutat 2004;23:630-631.
66. Pang T, Lei J, Zheng H, et al. [Prenatal diagnosis of oculocutaneous albinism type IV and discovery of a novel mutation]. Zhonghua Yi Xue Yi Chuan Xue Za Zhi 2011;28:1-5.
67. Park SH, Chae H, Kim Y, et al. Molecular analysis of Korean patients with oculocutaneous albinism. Jpn J Ophthalmol 2012;56:98-103
68. Park SK, Lee KH, Park KC, et al. Prevalent and novel mutations of the tyrosinase gene in Korean patients with tyrosinase-deficient oculocutaneous albinism. *Mol Cells*. 1997;7(2):187-191.
69. Passmore LA, Kaesmann-Kellner B, Weber BH. Novel and recurrent mutations in the tyrosinase gene and the P gene in the German albino population. Hum Genet 1999;105: 200-210.
70. Patrosso MC, Lando G, Penco S. Gene symbol: TYR. Disease: Albinism, oculocutaneous 1. Hum Genet 2008;124: 294.
71. Preising MN, Forster H, Gonser M, et al. Screening of TYR, OCA2, GPR143, and MC1R in patients with congenital nystagmus, macular hypoplasia, and fundus hypopigmentation indicating albinism. Mol Vis 2011;17: 939-948.
72. Qiu B, Ma T, Peng C, et al. Identification of Five Novel Variants in Chinese Oculocutaneous Albinism by Targeted Next-Generation Sequencing. Genetic testing and molecular biomarkers 2018; 22, 1-7.
73. Ray K, Chaki M, Mukhopadhyay A. Gene symbol: TYR. Disease: Albinism, oculocutaneous 1. Hum Genet 2005;117:299.
74. Ray K, Chaki M, Sengupta M. Novel human pathological mutations. Gene symbol: TYR. Disease: tyrosinase deficiency. Hum Genet 2007;122:555.
75. Rooryck C, Morice-Picard F, Elcioglu NH, et al. Molecular diagnosis of oculocutaneous albinism: new mutations in the OCA1-4 genes and practical aspects. Pigment Cell Melanoma Res 2008;21:583-587.
76. Rosenmann A, Bejarano-Achache I, Eli D, et al. Prenatal molecular diagnosis of oculocutaneous albinism (OCA) in a large cohort of Israeli families. Prenat Diagn 2009;29:939-946.
77. Rundshagen U, Zuhlke C, Opitz S, et al. Mutations in the MATP gene in five German patients affected by oculocutaneous albinism type 4. Hum Mutat 2004;23:106-110.
78. Saxena R, Verma IC. Novel human pathological mutations. Gene symbol: TYR. Disease: Albinism, oculocutaneous 1. Hum Genet 2010;127:488.
79. Schnur RE, Sellinger BT, Holmes SA, et al. Type I oculocutaneous albinism associated with a full-length deletion of the tyrosinase gene. J Invest Dermatol 1996;106:1137-1140.
80. Shah SA, Din SU, Raheem N, et al. Identification of a novel mutation (p.Ile198Thr) in gene TYR in a Pakistani family with nonsyndromic oculocutaneous albinism. *Clin Exp Dermatol*. 2014;39(5):646-648.
81. Shah SA, Raheem N, Daud S, et al. Mutational spectrum of the TYR and SLC45A2 genes in Pakistani families with oculocutaneous albinism, and potential founder effect of missense substitution (p.Arg77Gln) of tyrosinase. *Clin Exp Dermatol*. 2015;40(7):774-780.
82. Simeonov DR, Wang X, Wang C, et al. DNA Variations in Oculocutaneous Albinism: An Updated Mutation List and Current Outstanding Issues in Molecular Diagnostics. Hum Mutat. 2013; 34(6): 827–835.
83. Sengupta M, Chaki M, Arti N, et al. SLC45A2 variations in Indian oculocutaneous albinism patients. Mol Vis 2007;13:1406-1411.
84. Spritz RA. Molecular genetics of oculocutaneous albinism. Semin Dermatol 1993;12:167-172.
85. Spritz RA, Oh J, Fukai K, et al. Novel mutations of the tyrosinase (TYR) gene in type I oculocutaneous albinism (OCA1). Hum Mutat 1997;10:171-174.
86. Spritz RA, Strunk KM, Hsieh CL, et al. Homozygous tyrosinase gene mutation in an American black with tyrosinase-negative (type IA) oculocutaneous albinism. Am J Hum Genet 1991;48:318-324.
87. Straniero L, Rimoldi V, Soldà G, et al. Two novel splicing mutations in the SLC45A2 gene cause Oculocutaneous Albinism Type IV by unmasking cryptic splice sites. *J Hum Genet*. 2015;60(9):467-471.
88. Summers CG. Vision in albinism. Trans Am Ophthalmol Soc 1996;94:1095-1155.
89. Summers CG, Connett JE, Holleschau AM, et al. Does levodopa improve vision in albinism? Results of a randomized, controlled clinical trial. *Clin Exp Ophthalmol*. 2014;42(8):713-721.
90. Sun W, Shen Y, Shan S, et al. Identification of TYR mutations in patients with oculocutaneous albinism. *Mol Med Rep*. 2018;17(6):8409-8413.
91. Sundaresan P, Sil AK, Philp AR, et al. Genetic analysis of oculocutaneous albinism type 1 (OCA1) in Indian families: two novel frameshift mutations in the TYR Gene. Mol Vis 2004;10:1005-1010.
92. Takeda A, Tomita Y, Matsunaga J, et al. Molecular basis of tyrosinase-negative oculocutaneous albinism. A single base mutation in the tyrosinase gene causing arginine to glutamine substitution at position 59. J Biol Chem 1990;265:17792-17797.
93. Tomita Y, Takeda A, Okinaga S, et al. Human oculocutaneous albinism caused by single base insertion in the tyrosinase gene. Biochem Biophys Res Commun 1989;164: 990-996.
94. Tripathi RK, Bundey S, Musarella MA, et al. Mutations of the tyrosinase gene in Indo-Pakistani patients with type I (tyrosinase-deficient) oculocutaneous albinism (OCA). Am J Hum Genet 1993;53:1173-1179.
95. Tripathi RK, Strunk KM, Giebel LB, et al. Tyrosinase gene mutations in type I (tyrosinase-deficient) oculocutaneous albinism define two clusters of missense substitutions. Am J Med Genet 1992;43:865-871.
96. Tsai CH, Tsai FJ, Wu JY, et al. Insertion/deletion mutations of type I oculocutaneous albinism in chinese patients from Taiwan. Hum Mutat 1999;14:542.
97. Urtatiz O, [Sanabria](https://pubmed.ncbi.nlm.nih.gov/?term=Sanabria+D&cauthor_id=25455140) D, [Lattig](https://pubmed.ncbi.nlm.nih.gov/?term=Lattig+MC&cauthor_id=25455140) MC. Oculocutaneous Albinism (OCA) in Colombia: First Molecular Screening of the TYR and OCA2 Genes in South America. J Dermatol Sci. 2014;76(3):260-262.
98. Verhagen JM, Huijmans JG, Williams M, et al. Incidental finding of alpha-methylacyl-CoA racemase deficiency in a patient with oculocutaneous albinism type 4. Am J Med Genet A 2012(11); 2931-2934.
99. [Vidal-Ríos](https://pubmed.ncbi.nlm.nih.gov/?term=Vidal-R%C3%ADos+P&cauthor_id=23085315) P, [Fernández-Seara](https://pubmed.ncbi.nlm.nih.gov/?term=Fern%C3%A1ndez-Seara+MJ&cauthor_id=23085315) MJ, [Cortés](https://pubmed.ncbi.nlm.nih.gov/?term=Cort%C3%A9s+E&cauthor_id=23085315) E, et al. [Oculocutaneous Albinism 1B Associated With a New Mutation in the TYR Gene]. An Pediatr (Barc). 2013; 78(5):339-340.
100. Wang Y, Guo X, Li W, et al. Four novel mutations of TYR gene in Chinese OCA1 patients. J Dermatol Sci 2009;53:80-81.
101. [Wang](https://www.ncbi.nlm.nih.gov/pubmed/?term=Wang%20Y%5BAuthor%5D&cauthor=true&cauthor_uid=25919014) Y, [Wang](https://www.ncbi.nlm.nih.gov/pubmed/?term=Wang%20Z%5BAuthor%5D&cauthor=true&cauthor_uid=25919014) Z, [Chen](https://www.ncbi.nlm.nih.gov/pubmed/?term=Chen%20M%5BAuthor%5D&cauthor=true&cauthor_uid=25919014) M, et al. Mutational Analysis of the TYR and OCA2 Genes in Four Chinese Families with Oculocutaneous Albinism. [PLoS One](https://www.ncbi.nlm.nih.gov/pmc/articles/PMC4412409/). 2015; 10(4): e0125651.
102. [Wang](https://pubmed.ncbi.nlm.nih.gov/?term=Wang+Y&cauthor_id=30341532) Y, [Yi-Fan Zhou](https://pubmed.ncbi.nlm.nih.gov/?term=Zhou+YF&cauthor_id=30341532) YF, [Na She](https://pubmed.ncbi.nlm.nih.gov/?term=Shen+N&cauthor_id=30341532)n N, et al. 
     Identification of a Homozygous Missense Mutation in the TYR Gene in a Chinese Family with OCA1. Curr Med Sci 2018 Oct;38(5):932-936.
103. Wei A, Wang Y, Long Y, et al. A comprehensive analysis reveals mutational spectra and common alleles in Chinese patients with oculocutaneous albinism. J Invest Dermatol 2010;130:716-724.
104. Wei A, Yang X, Lian S, et al. Implementation of an optimized strategy for genetic testing of the Chinese patients with oculocutaneous albinism. J Dermatol Sci 2011;62:124-127.
105. [Wei](https://pubmed.ncbi.nlm.nih.gov/?term=Wei+AH&cauthor_id=23324268) AH, [Yang](https://pubmed.ncbi.nlm.nih.gov/?term=Yang+XM&cauthor_id=23324268) XM, [Lian](https://pubmed.ncbi.nlm.nih.gov/?term=Lian+S&cauthor_id=23324268) S, et al. Genetic Analyses of Chinese Patients With Digenic Oculocutaneous Albinism**.** Chin Med J (Engl). 2013;126(2):226-230
106. Wu Q, Shi HR, Liu N, et al. [Early prenatal genetic diagnosis of oculocutaneous albinism type I in seven families]. Zhonghua Yi Xue Yi Chuan Xue Za Zhi 2012;29:377-381.
107. Xu B, Pang T, Yao CQ, et al. [Identification of a novel pathogenic mutation in MATP gene with oculocutaneous albinism type IV from a consanguineous marriage family]. Zhonghua Yi Xue Za Zhi 2012;92:254-258.
108. Yang Q, Yi S, Li M, et al. Genetic analyses of oculocutaneous albinism types 1 and 2 with four novel mutations. *BMC Med Genet*. 2019;20(1):106.
109. Zahed L, Zahreddine H, Noureddine B, et al. Molecular basis of oculocutaneous albinism type 1 in Lebanese patients. J Hum Genet 2005;50:317-319.
110. Zheng H, Huang ZG, Wen RQ, Li HY. Zhongguo Ying Yong Sheng Li Xue Za Zhi. 2011;27(3):329-332.
